# Supplementary material for: The role of ATP synthase subunit e (ATP5I) in mediating the metabolic and antiproliferative effects of metformin in cancer cells
Source: eLife. 2026 May 15;13:RP102680. doi: 10.7554/eLife.102680 (PMC13179060; doi:10.7554/eLife.102680)
Supplement: Figure 1—source data 1. [file elife-102680-fig1-data1.docx]

**Figure 1A – Synthesis**

**2,5-Dioxopyrrolidin-1-yl 5-((3aS,4S,6aR)-2-oxo-hexahydro-1H-thieno[3,4-d]imidazol-4-yl)pentanoate**

Biotin (3.00 g, 12.3 mmoles), EDC (2.82 g, 14.7 mmoles) and NHS (1.70 g, 14.7 mmoles) were dissolved in 100 mL of DMF and the reaction mixture was stirred at room temperature overnight. The suspension was then concentrated under reduced pressure and the residue was triturated with EtOH:AcOH:H_2_O (95:1:4). The biotin-NHS (**1**) (3.40 g, 81% yield) was obtained as a white powder.

**^1^H NMR (400 MHz, DMSO-*d_6_*)** δ: 6.43 (s, 1H), 6.37 (s, 1H), 4.30-4.32 (m, 1H), 4.14-4.17 (m, 1H), 3.09-3.13 (m, 1H), 2.82-2.86 (m, 5H), 2.68 (t, 2H, *J* = 7.2 Hz), 2.58 (d, 1H, *J* = 12.4), 1.61-1.68 (m, 3H), 1.39-1.54 (m, 3H)

**^13^C NMR (400 MHz, DMSO*-d_6_*)** δ: 170.6, 169.2, 163.0, 61.3, 59.5, 55.5, 30.3, 28.1, 27.9, 25.7, 24.6

**HRMS**: m/z calcd for C_14_H_19_N_3_O_5_S [M+H]^+^: 342.1045, found: 342.1126

***N*-(6-Aminohexyl)imidodicarbonimidic diamide chloride**

N-Boc-1,6-hexanediamine (1 mL, 4.62 mmol) and dicyandiamide (389 mg, 4.62 mmol) and chlorotrimethylsilane (650 mL, 5.09 mmol) were dissolved in 10 mL of MeCN and the reaction was stirred at 160°C for 3 h. After the mixture was cooled to approximately 50°C, 4 mL of isopropanol (iPrOH) was added slowly until precipitation. The precipitate was then filtered and washed twice with MeCN to obtain 6-aminohexylbiguanide hydrochloride (**2**) (1.05 g, 76 % yield) as white powder.

**^1^H NMR (500 MHz, MeOD)** δ: 3.36 (s, 2H), 2.95 (s, 2H), 1.71 (s, 4H), 1.48 (s, 4H)

**^13^C NMR (500 MHz, MeOD)** δ: 156.6, 154.0, 44.1, 40.7, 28.6, 28.4, 27.3, 27.0

**HRMS**: m/z calcd for C_8_H_20_N_6_  [M + H]^+^: 201.18222, found: 201.18152

**N-(5-(3a*S*,4*S*,6a*R*)-2-oxo-hexahydro-1*H*-thieno[3,4-*d*]imidazole-4 yl)pentanamido)hexylimido-dicarbonimidic diamide chloride**

6-aminohexylbiguanide chloride salt (**2**) (300 mg, 1.5 mmol ) was dissolved in 6 mL of DMF and N,N-Diisopropylethylamine (650 mL, 3.74 mmol) was added dropwise. Then, biotin-NHS (**1**) (426 mg, 1.25 mmol) was added and the reaction mixture was stirred at room temperature overnight. The suspension was then concentrated under reduced pressure to finally obtain an orange oil. The suspension oil was then solubilized in 1 mL of MeOH and purified using a preparative liquid chromatography. The corresponding fractions were concentrated under reduced pressure and frieze-dried overnight to obtain the biotin functionalized biguanide hydrochloride (**3**) as a white volatile powder (240 mg, 45 % of yield).

**^1^H NMR (500 MHz, D_2_O)** δ: 4.60-4.64 (m, 1H), 4.42-4.45 (m, 1H), 3.32-3.37 (m, 1H), 3.14-3.25 (m, 4H), 3.01 (dd, 1H, *J* = 13 Hz, *J* = 5 Hz), 2.79 (d, 1H, *J* = 13 Hz), 2.26 (t, 2H, *J* = 7.2 Hz), 1.48-1.78 (m, 8H), 1.28-1.47 (m, 6H)

**^13^C NMR (500 MHz, D_2_O)** δ: 176.6, 170.9, 165.3, 159.9, 62.1, 60.2, 55.4, 39.7, 39.1, 35.5, 28.2, 27.8, 27.7, 25.6, 25.5, 25.2

**HRMS**: m/z calcd for C_18_H_34_N_8_O_2_S [M+H]^+^: 427.26, found: 427.2588

**LC-MS purity**: > 99.5 % (Acq method, LC_0_50_15min_MeOH, column Atlantis dC18, 2.1 x 100 mm, 3μ, t_r_ = 6.7 min)

***N*-[6-[(1,1-Dimethylethoxy)carbonyl]aminohexyl]-5-(3a*S*,4*S*,6a*R*)-2-oxo-hexahydro-1*H*-thieno[3,4-*d*]imidazole-4-yl)pentanamide**

Biotin-NHS (**1**) (1.5 g, 4.39 mmoles) and N-Boc-1,6-hexanediamine (1.18 mL, 5.27 mmol) were dissolved in 60 mL of DMF and the reaction mixture was stirred at room temperature overnight. The suspension was then concentrated under reduced pressure to obtain a yellow solid. The residue was solubilized in 2 mL of MeOH and purified using normal silica gel column chromatography (0-10 % MeOH in DCM) to obtain **4** (1.60 g, 82 % of yield) as a white solid.

**^1^H NMR (500 MHz, CD_3_OD**) δ: 4.50-4.53 (m, 1H), 4.30-4.34 (m, 1H), 3.16-3.25 (m, 3H), 3.04 (t, 2H, *J* = 7 Hz), 2.96 (dd, 1H, *J* = 12.5 Hz, *J* = 5 Hz), 2.73 (d, 1H, *J* = 13 Hz), 2.21 (t, 2H, *J* = 7.3 Hz), 1.57-1.80 (m, 4H), 1.45-1.54 (m, 15H), 1.29-1.42 (m, 4H)

**^13^C NMR (500 MHz, CD_3_OD)** δ: 175.9, 166, 158.5, 79.7, 63.3, 61.5, 56.9, 41.1, 40.9, 40.2, 36.7, 30.8, 29.7, 29.4, 28.7, 27.5, 27.4, 26.8

**HRMS**: m/z calcd for C_21_H_38_N_4_O_4_S [M+H]^+^: 443.2614, found: 443.2714

***N*-(6-Aminohexyl)-5-(3a*S*,4*S*,6a*R*)-2-oxo-hexahydro-1*H*-thieno[3,4-*d*]imidazole-4-yl)pentanamide chloride**

Compound **4** (180 mg, 407 mmol) was solubilized in 10 mL of MeOH, then 100 μL of a solution of HCl gas in dry MeOH (3 M) was added and the reaction mixture was stirred at room temperature overnight. The mixture was then concentrated under reduced pressure to give a translucid oil that was freeze-dried to obtain the biotin functionalized amine hydrochloride (**5)** (130 mg, 93 % of yield) as a white solid.

**^1^H NMR (500 MHz, CD_3_OD)** δ: 4.55-4.57 (m, 1H), 4.35-4.38 (m, 1H), 3.20-3.24 (m, 1H), 3.15 (t, 2H, *J* = 7 Hz), 2.92 (dd, 1H, *J* =13 Hz, *J* = 5 Hz), 2.87 (t, 2H, *J* = 7.8 Hz), 2.71 (d, 2H, *J* = 13 Hz), 2.21 (t, 2H, *J* = 7.2 Hz), 1.47-1.74 (m, 8H), 1.30-1.43 (m, 6H)

**^13^C NMR (500 MHz, CD_3_OD**) δ: 176.6, 166.0, 64.3, 62.7, 57.1, 40.9, 40.8, 40.6, 36.6 30.2, 29.9, 29.6, 28.6, 27.5, 27.2, 27.1

**HRMS**: m/z calcd for C_16_H_30_N_4_O_2_S [M+H]^+^: 343.21622, found: 343.21667

**LC-MS purity:** >99.5 (Acq method, LC_5_70_15min_MeOH, HPLC column Phenomenex Synergi POLAR-RP 150 x 4.6 mm, 4µm, t_r_ = 7.8min)


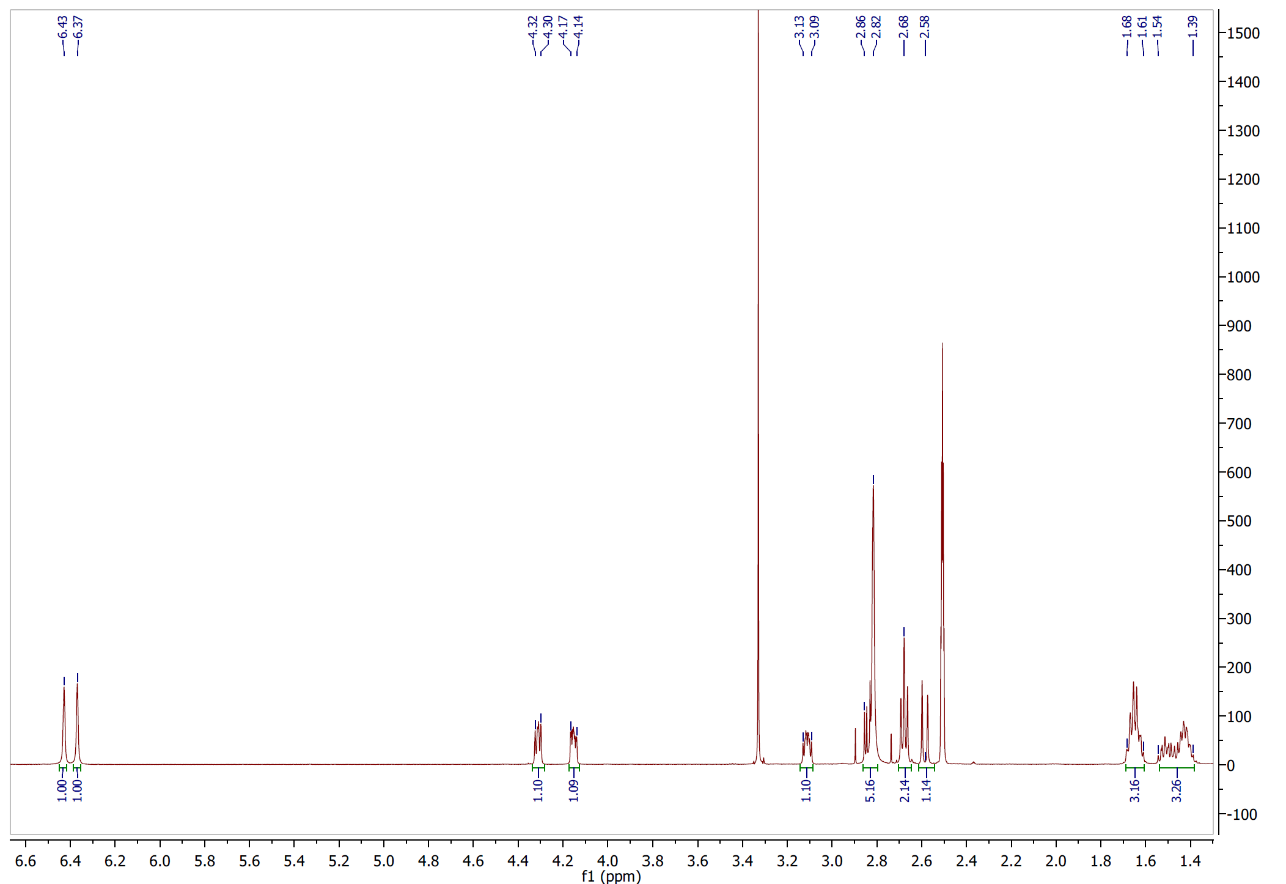

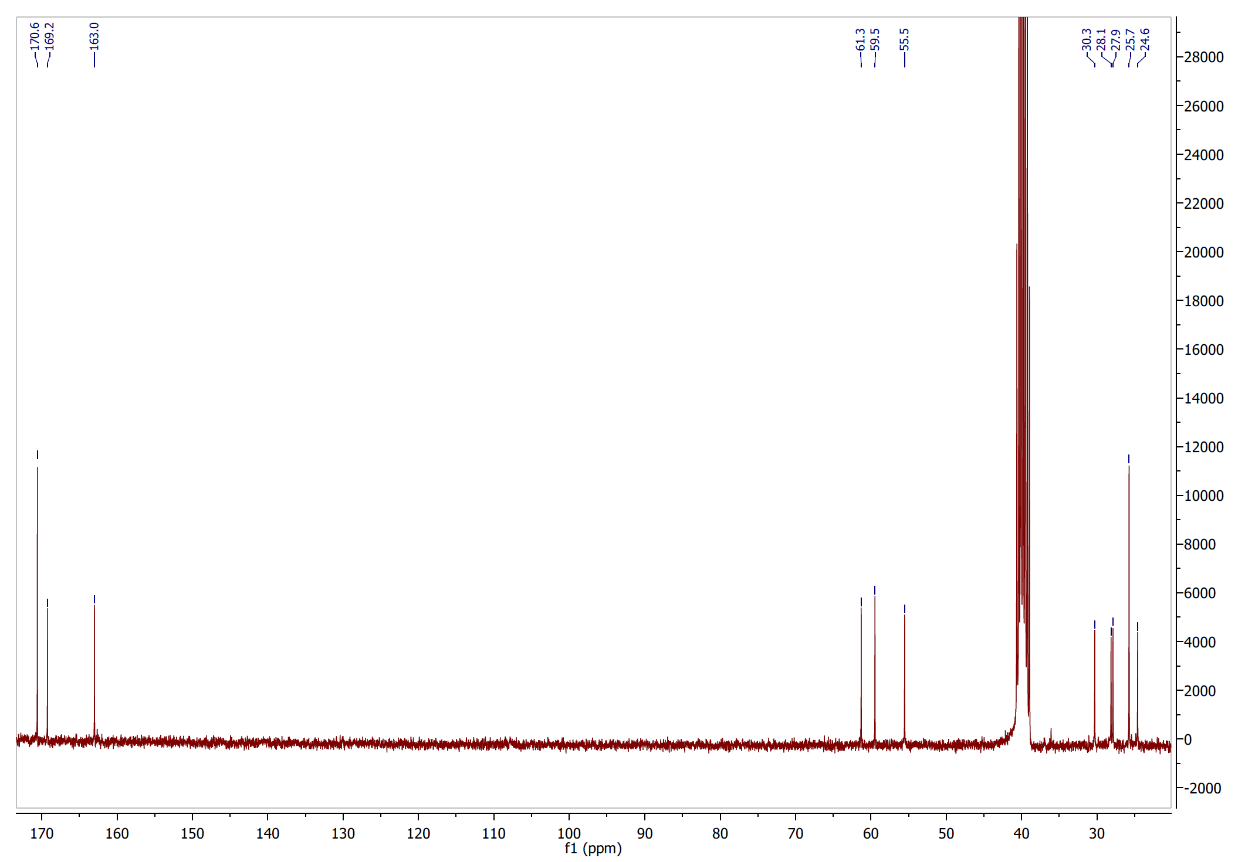

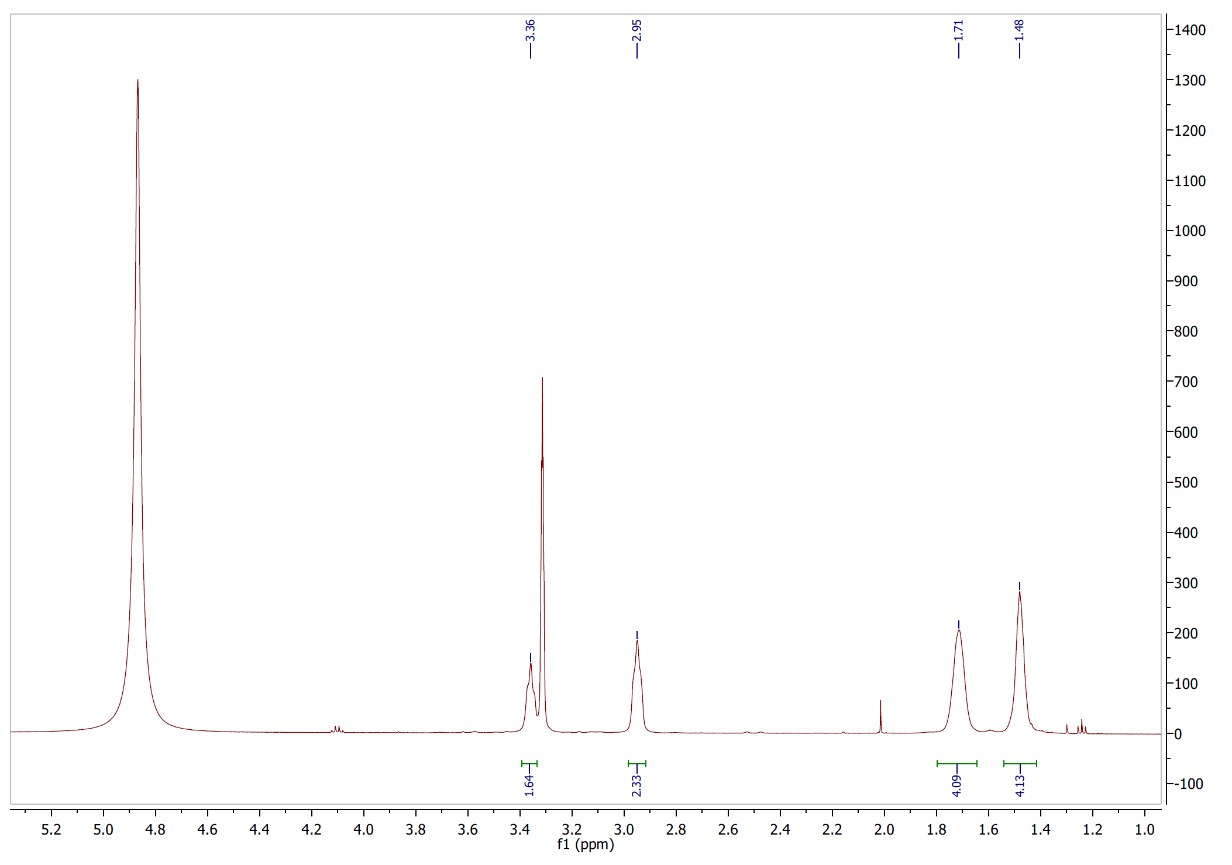


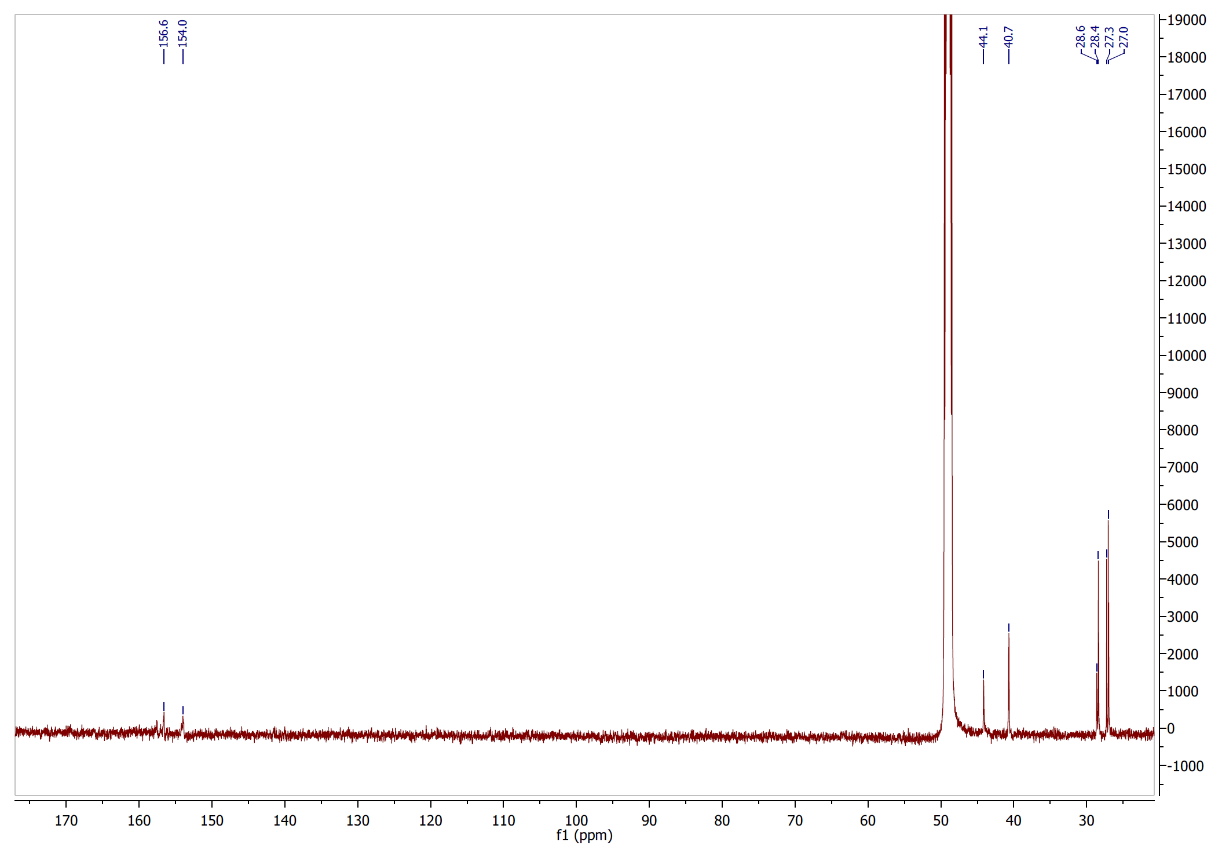

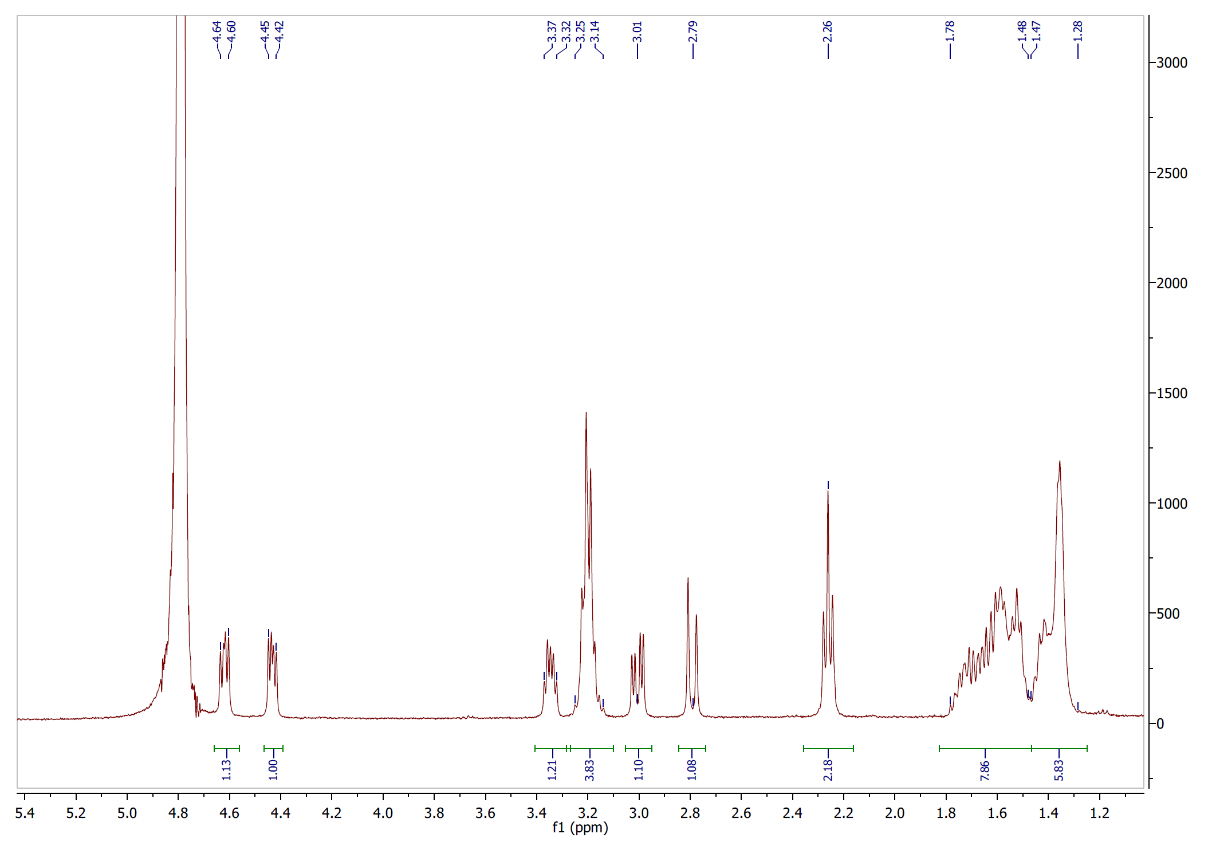

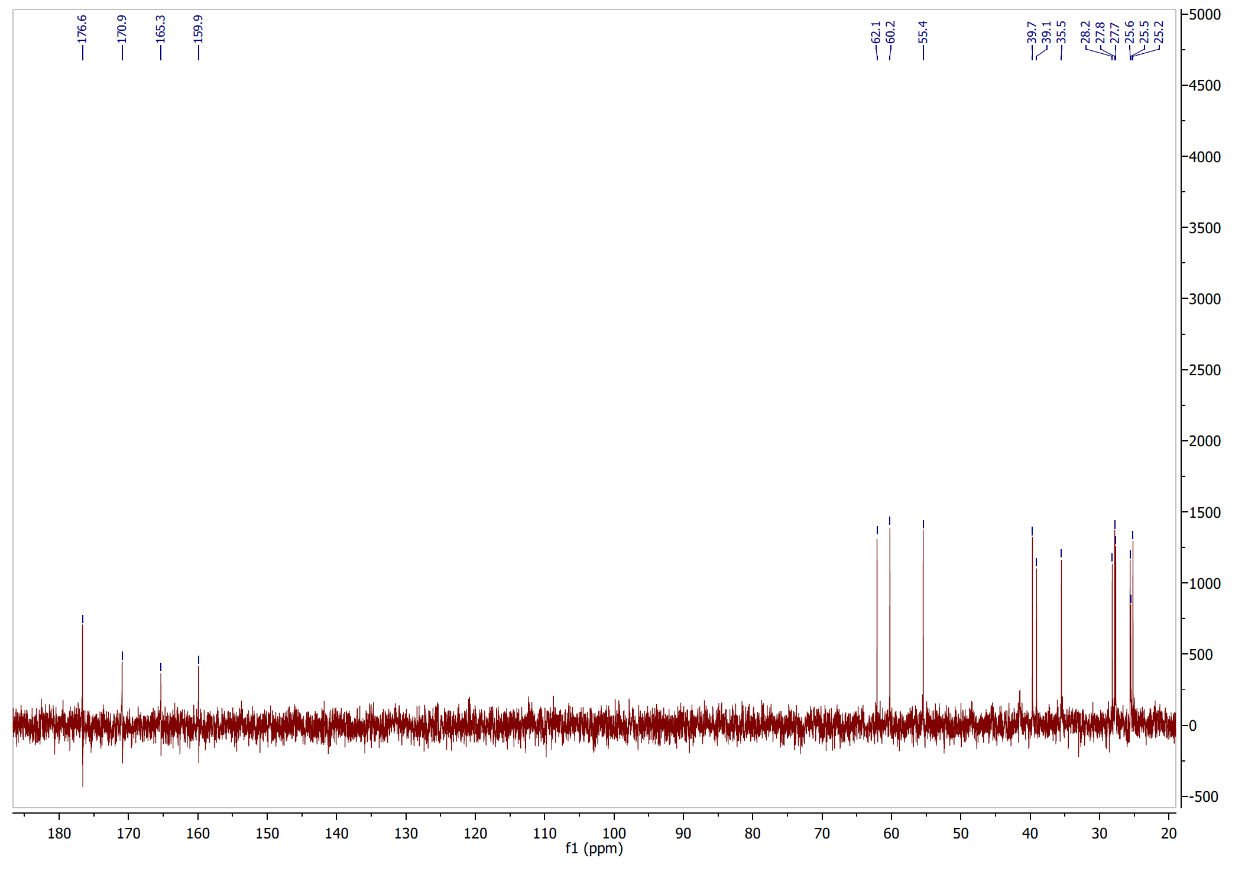

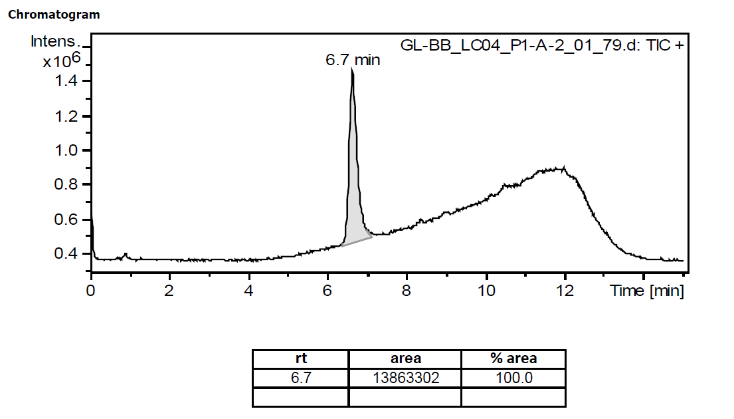

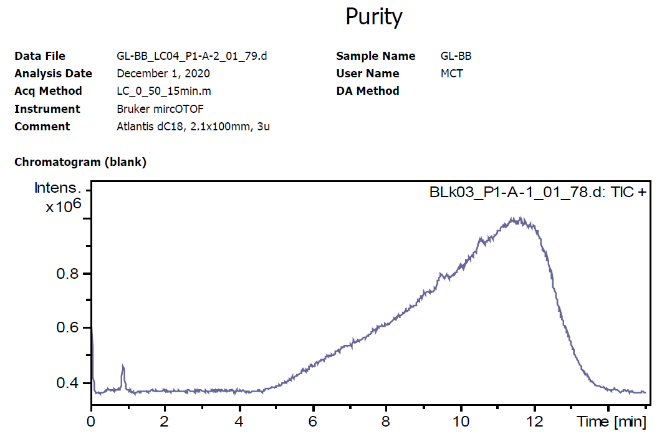


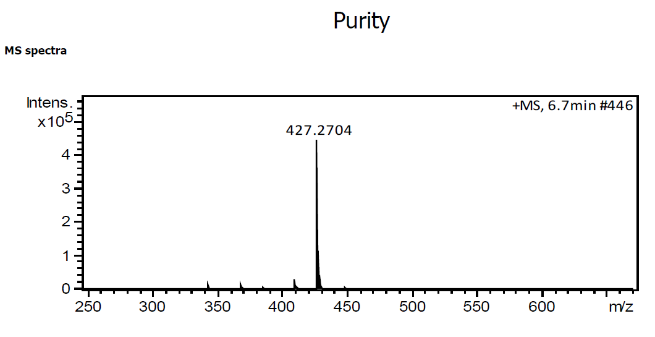


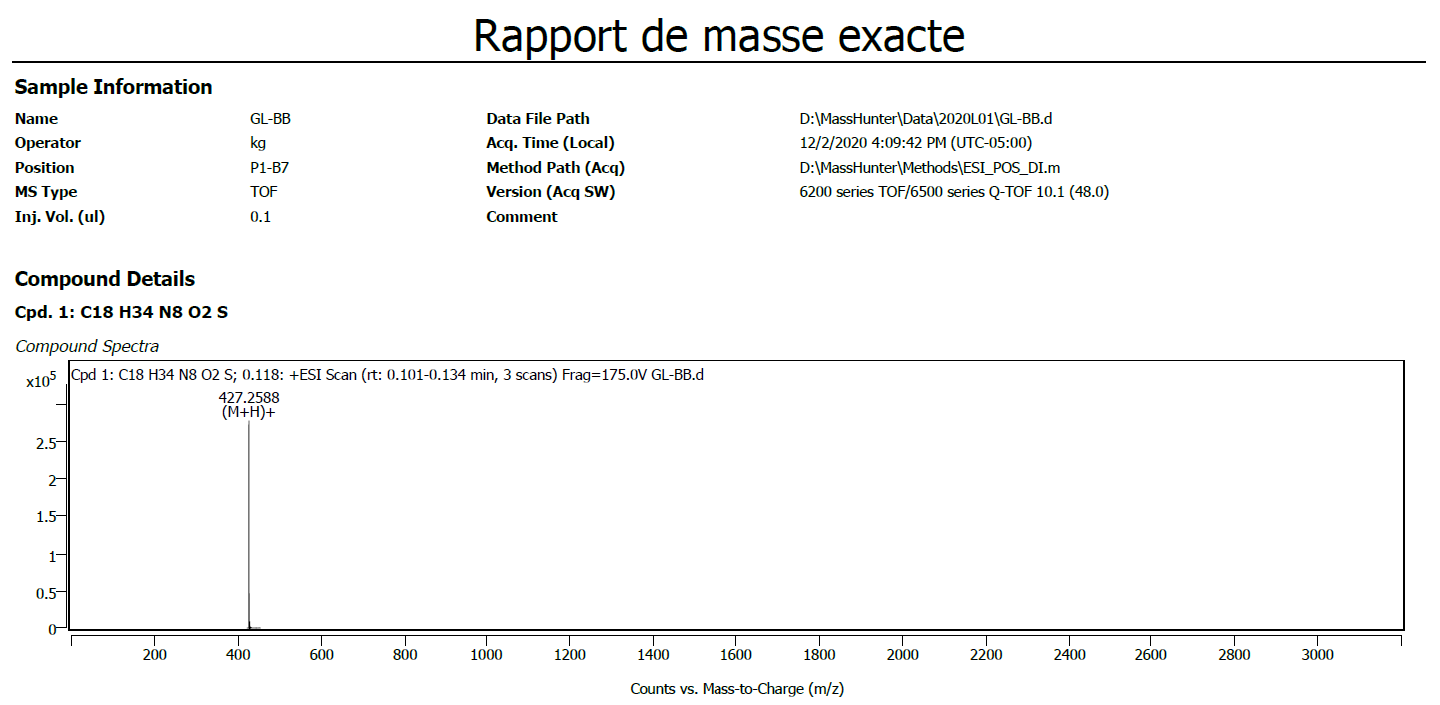


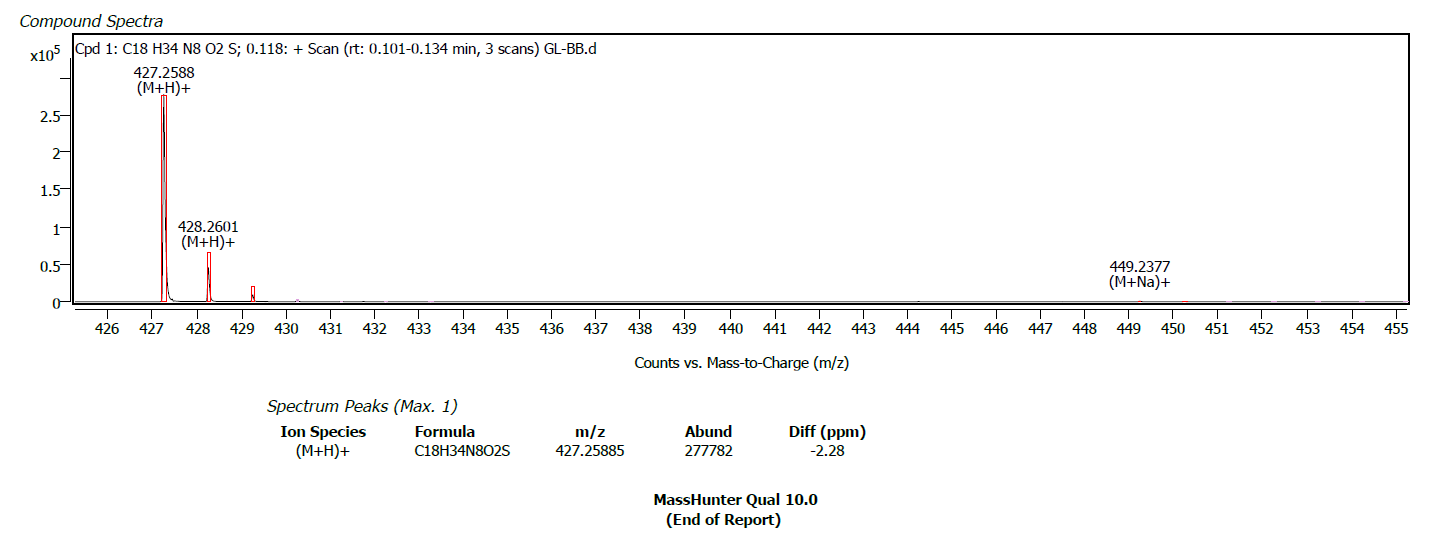


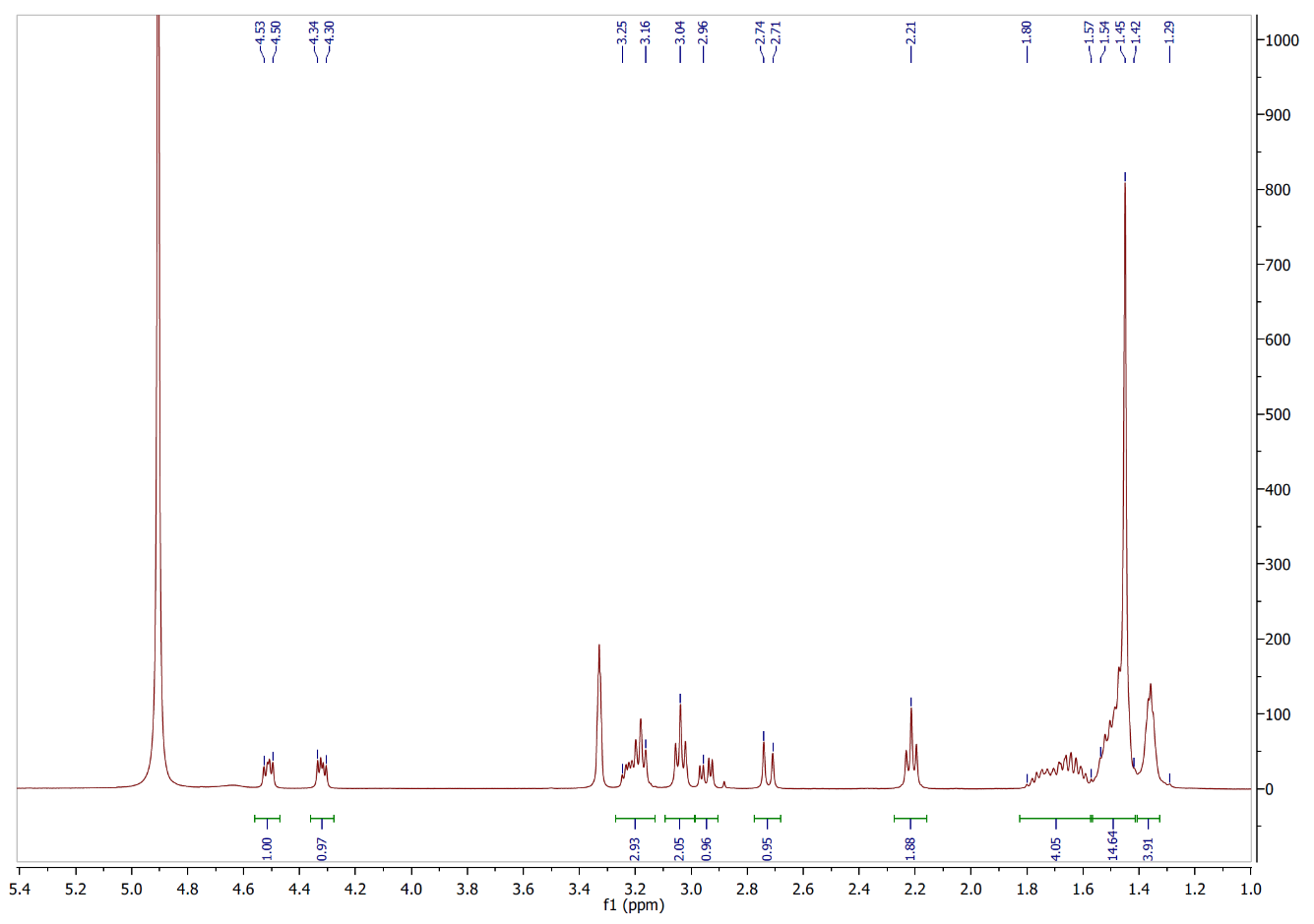

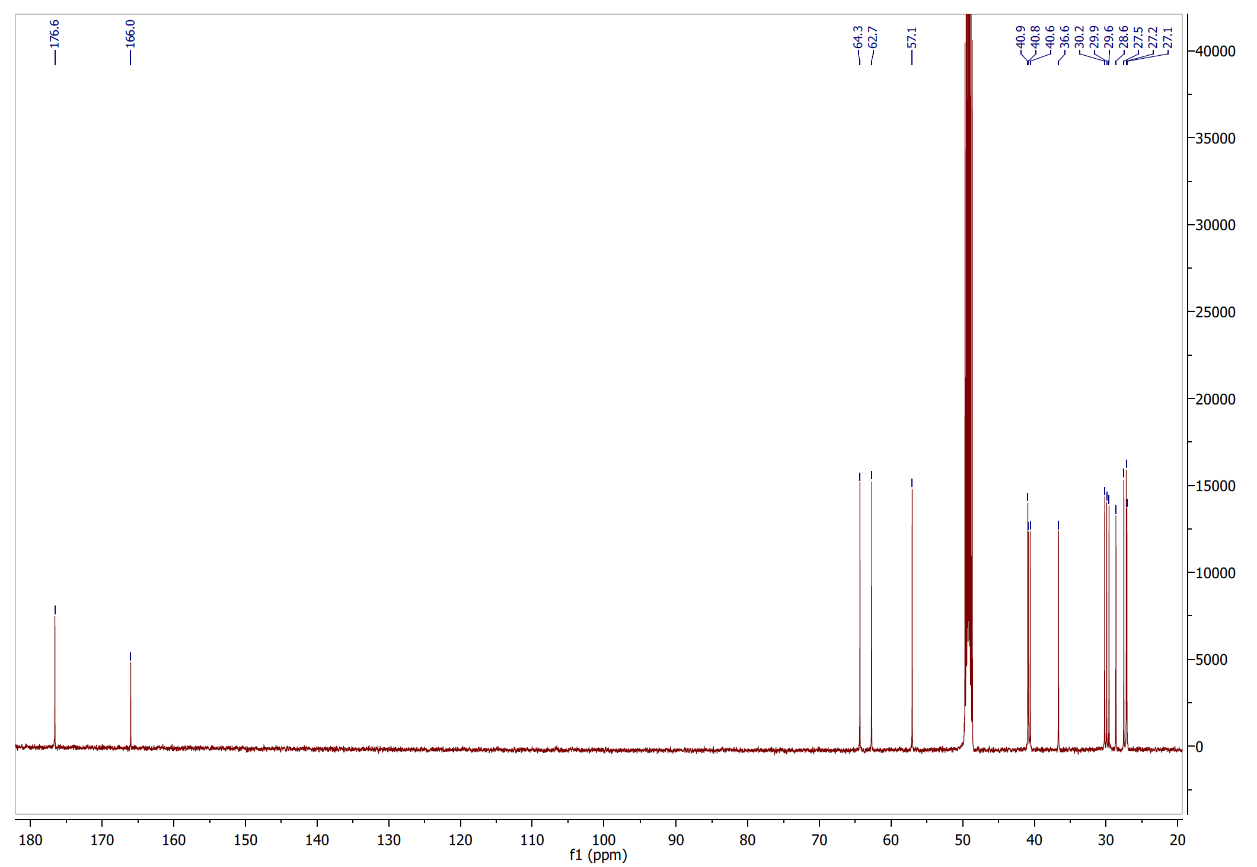

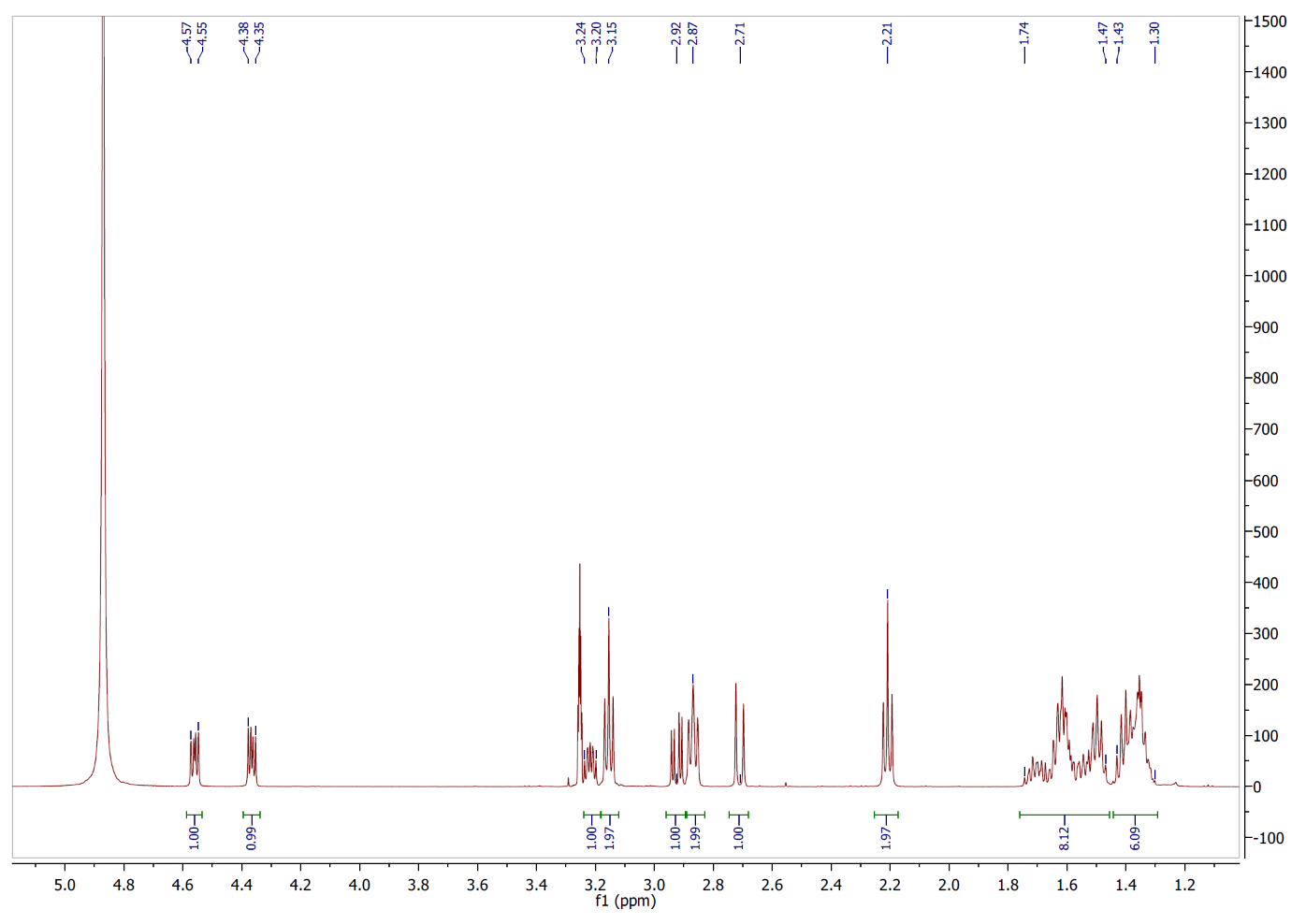

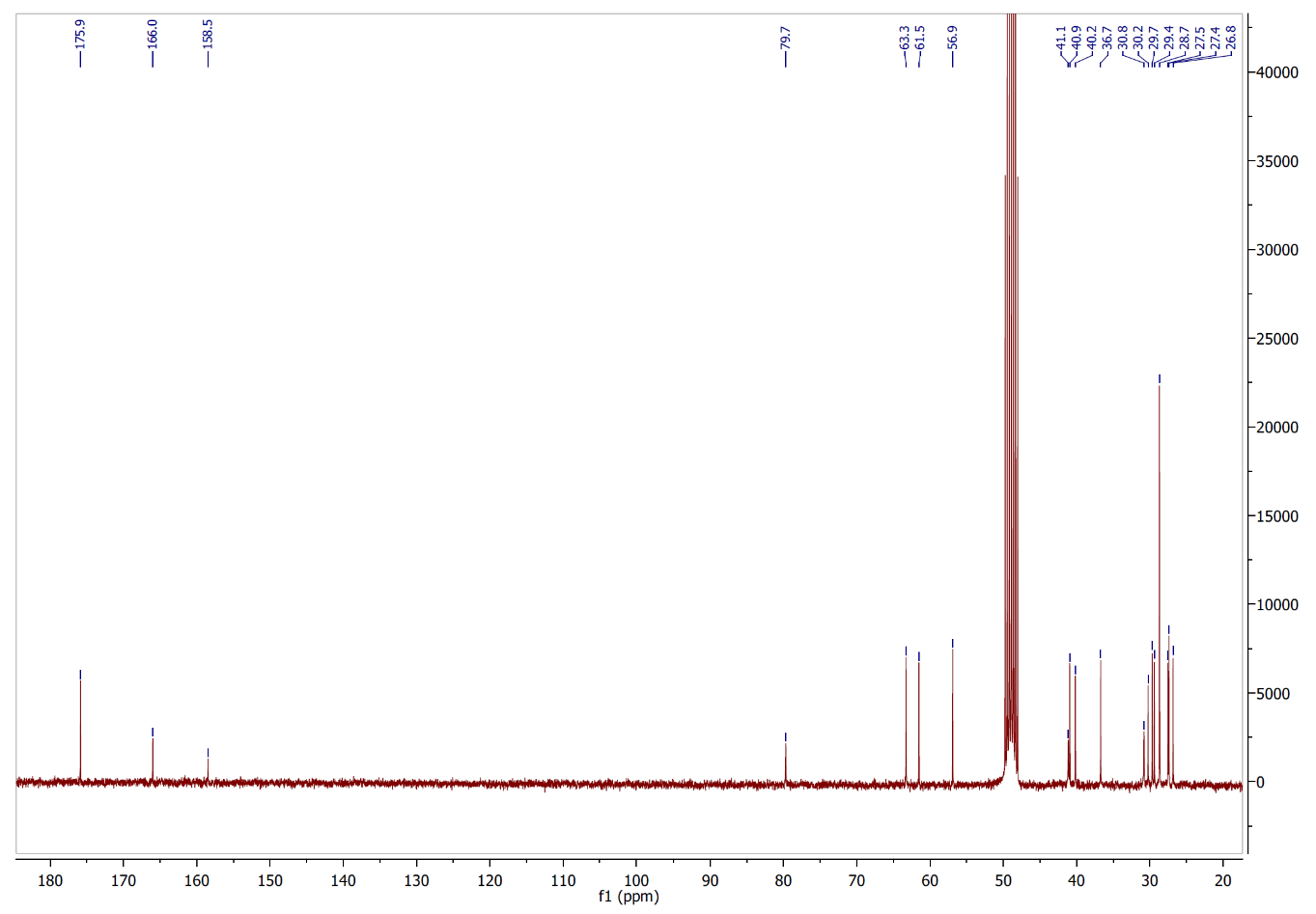

**
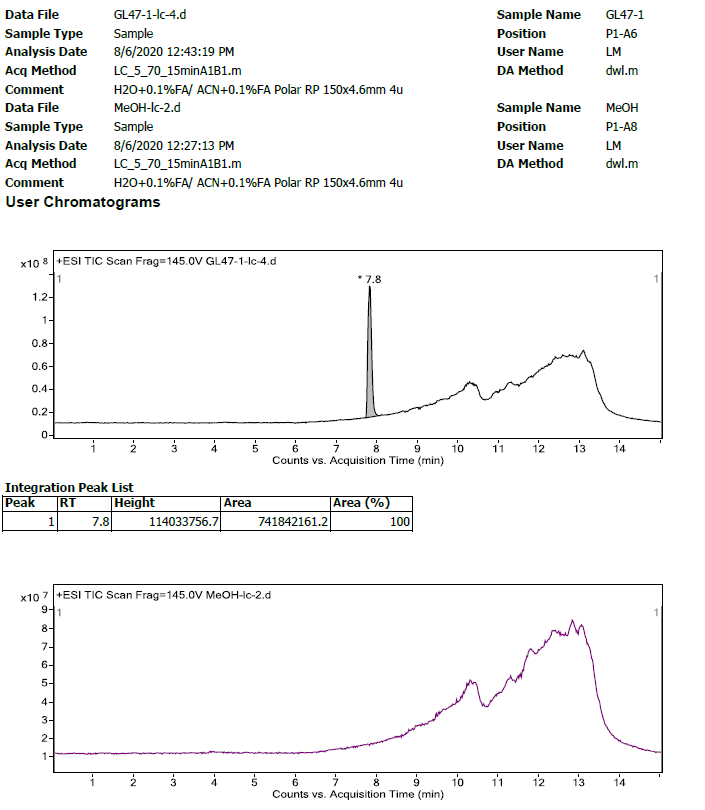

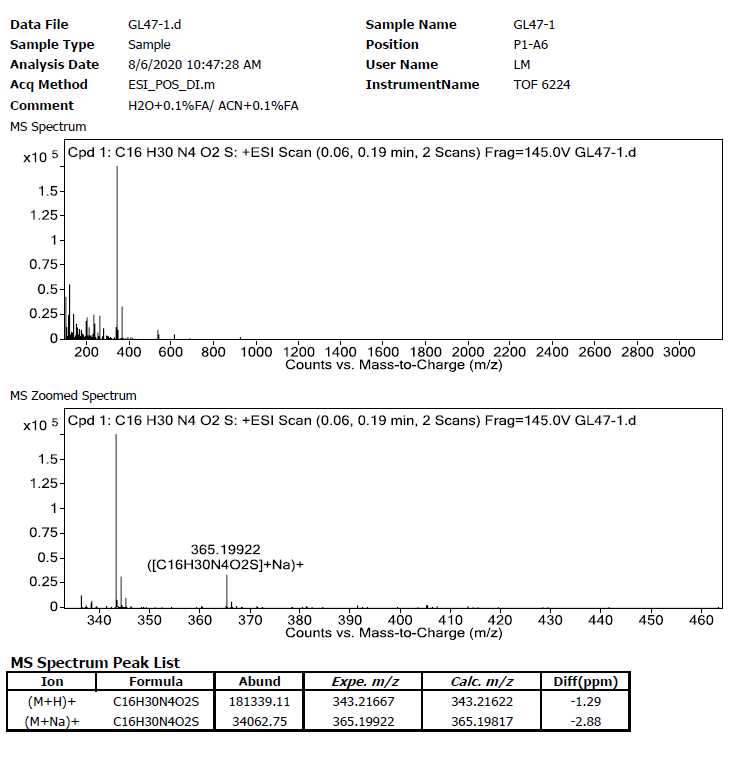
**
