# Supplementary material for: The role of ATP synthase subunit e (ATP5I) in mediating the metabolic and antiproliferative effects of metformin in cancer cells
Source: eLife. 2026 May 15;13:RP102680. doi: 10.7554/eLife.102680 (PMC13179060; doi:10.7554/eLife.102680)
Supplement: Figure 1—source data 2. — Many of our source data contains cut blotting membranes. Blotting membranes were cut after transfer to allow probing for proteins with different molecular weights. Each membrane section was incubated with the appropriate antibody corresponding to the target protein size. [file elife-102680-fig1-data2.zip › Figure 1 - Source data 2/Figure 1B_Source data 2.pdf]

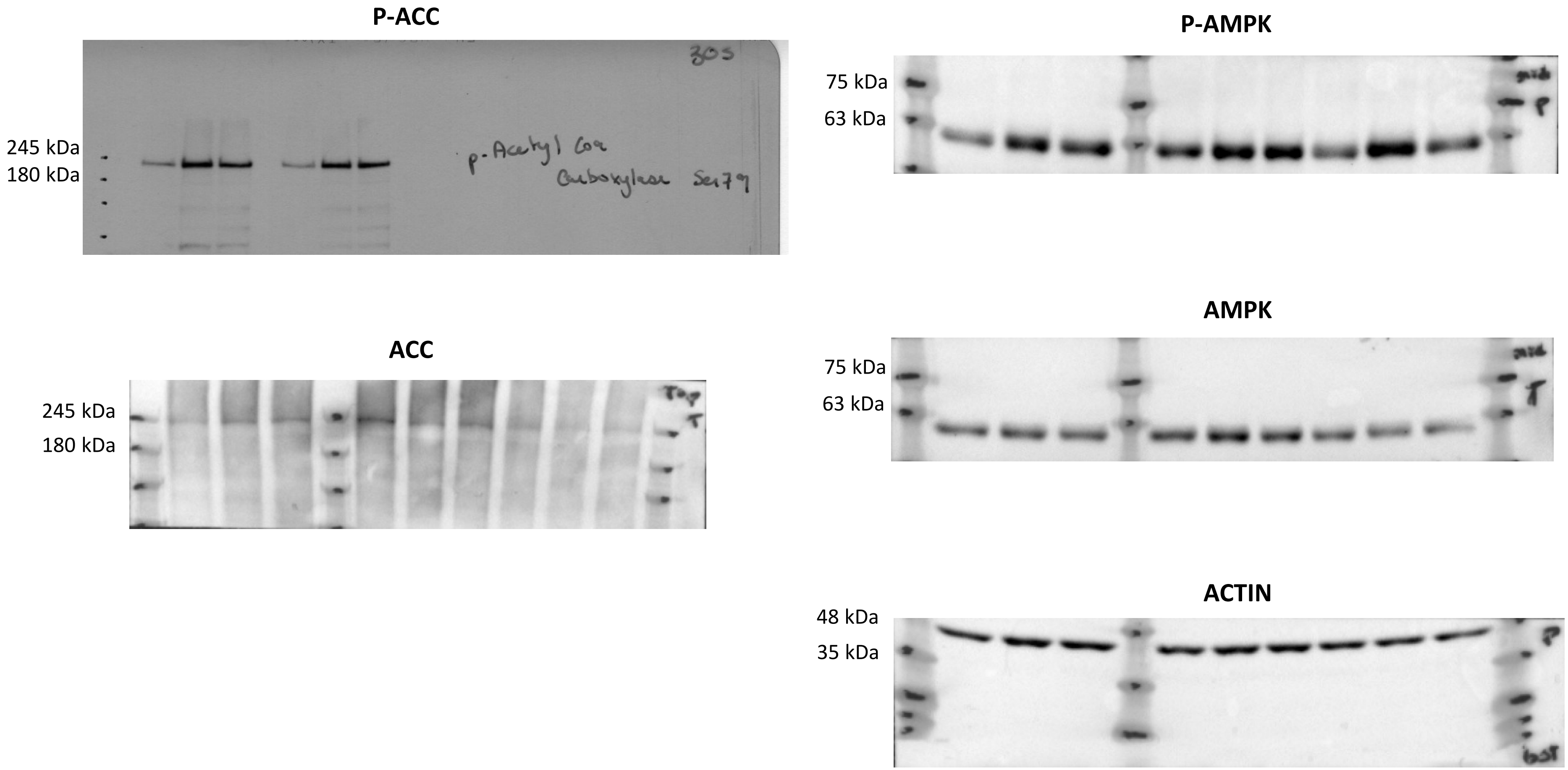

Figure 1B, Source Data 2. Original membranes corresponding to Figure 1B. Lanes 1–3 correspond to N = 1 for untreated (control) cells, metformin-treated cells, and biotin-functionalized biguanide-treated cells, respectively. Lanes 4–6 correspond to N = 2 under the same conditions. Lanes 7–9 for ACC, P-AMPK, AMPK, and ACTIN correspond to N = 3 under the same conditions. All experimental conditions are annotated on the corresponding membranes. Apparent molecular weight positions are manually annotated to indicate the size of the detected bands.
