# Supplementary material for: The role of ATP synthase subunit e (ATP5I) in mediating the metabolic and antiproliferative effects of metformin in cancer cells
Source: eLife. 2026 May 15;13:RP102680. doi: 10.7554/eLife.102680 (PMC13179060; doi:10.7554/eLife.102680)
Supplement: Figure 1—source data 2. — Many of our source data contains cut blotting membranes. Blotting membranes were cut after transfer to allow probing for proteins with different molecular weights. Each membrane section was incubated with the appropriate antibody corresponding to the target protein size. [file elife-102680-fig1-data2.zip › Figure 1 - Source data 2/Figure 1F_Source data 2.pdf]

ATP5I

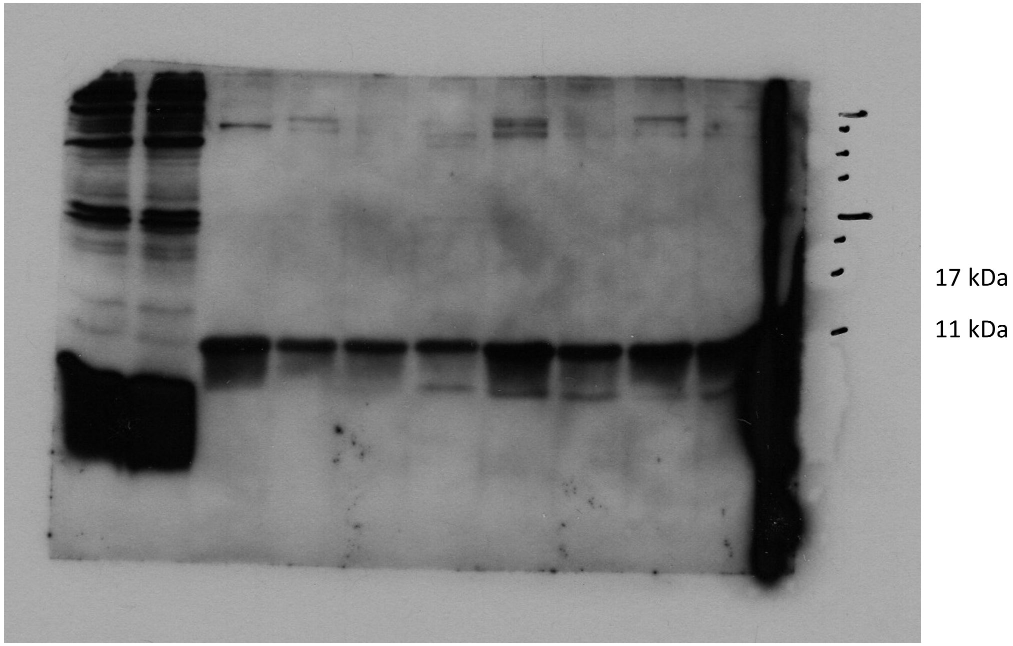

Figure 1F, Source Data 2. Original membrane corresponding to Figure 1F. Lane 1 represents whole cell lysate from non-transfected cells. Lane 2 corresponds to whole cell lysate from pcDNA-ATP5I-transfected cells. Lanes 3–6 correspond to pull-down incubations performed in non-transfected conditions with beads alone, biotin, biotin-functionalized amine, and biotin-functionalized biguanide, respectively. Lanes 7–10 correspond to the same incubation conditions performed in pcDNA-ATP5I-transfected cells. This membrane was acquired from autoradiography film, and the molecular weight marker positions are annotated manually to indicate the apparent size of the detected bands.
