# Supplementary figures and images for: The role of ATP synthase subunit e (ATP5I) in mediating the metabolic and antiproliferative effects of metformin in cancer cells
Source: eLife. 2026 May 15;13:RP102680. doi: 10.7554/eLife.102680 (PMC13179060; doi:10.7554/eLife.102680)

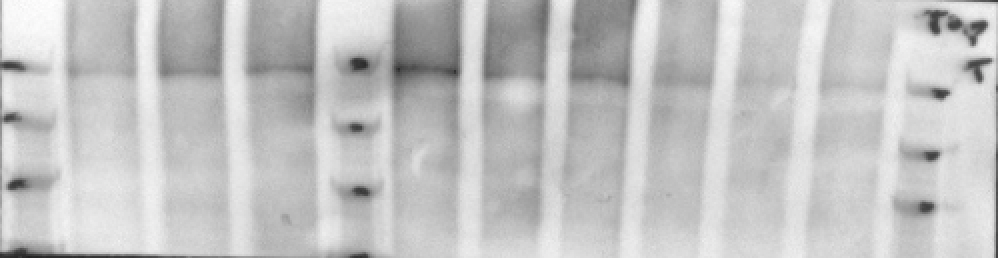

Supplement: Figure 1—source data 3. [file elife-102680-fig1-data3.zip › Figure 1 - Source data 3/Figure 1B_ Source data 3/ACC.png]

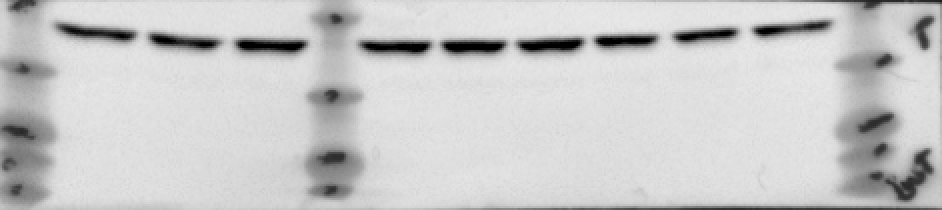

Supplement: Figure 1—source data 3. [file elife-102680-fig1-data3.zip › Figure 1 - Source data 3/Figure 1B_ Source data 3/ACTIN.png]

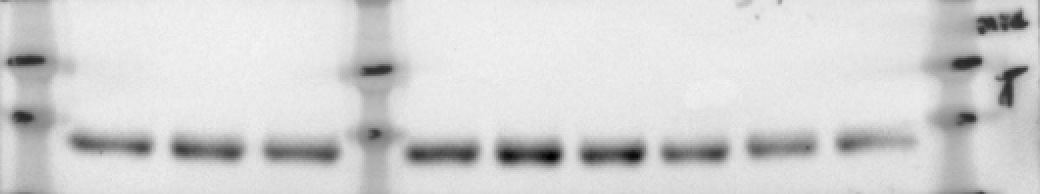

Supplement: Figure 1—source data 3. [file elife-102680-fig1-data3.zip › Figure 1 - Source data 3/Figure 1B_ Source data 3/AMPK.png]

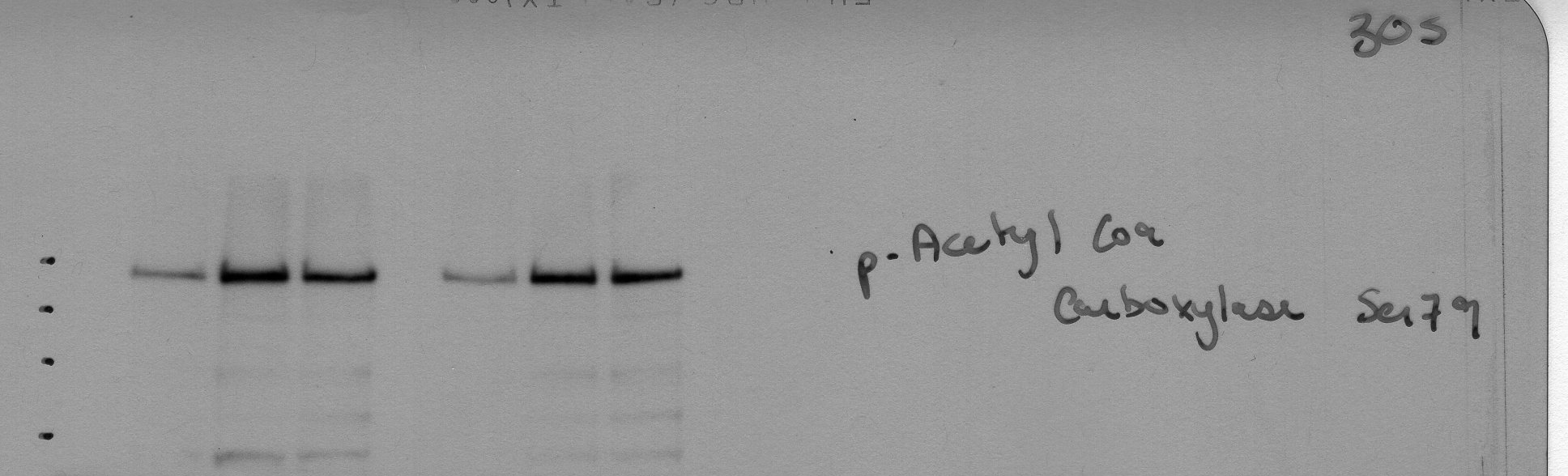

Supplement: Figure 1—source data 3. [file elife-102680-fig1-data3.zip › Figure 1 - Source data 3/Figure 1B_ Source data 3/P-ACC.tif]

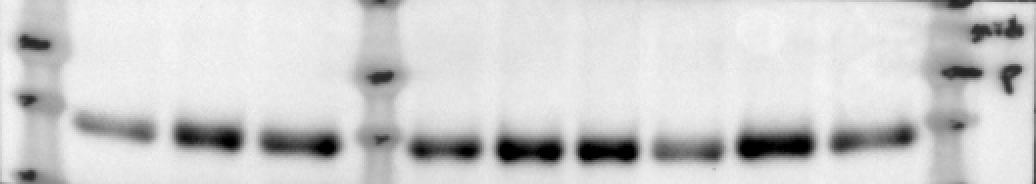

Supplement: Figure 1—source data 3. [file elife-102680-fig1-data3.zip › Figure 1 - Source data 3/Figure 1B_ Source data 3/pAMKP.png]

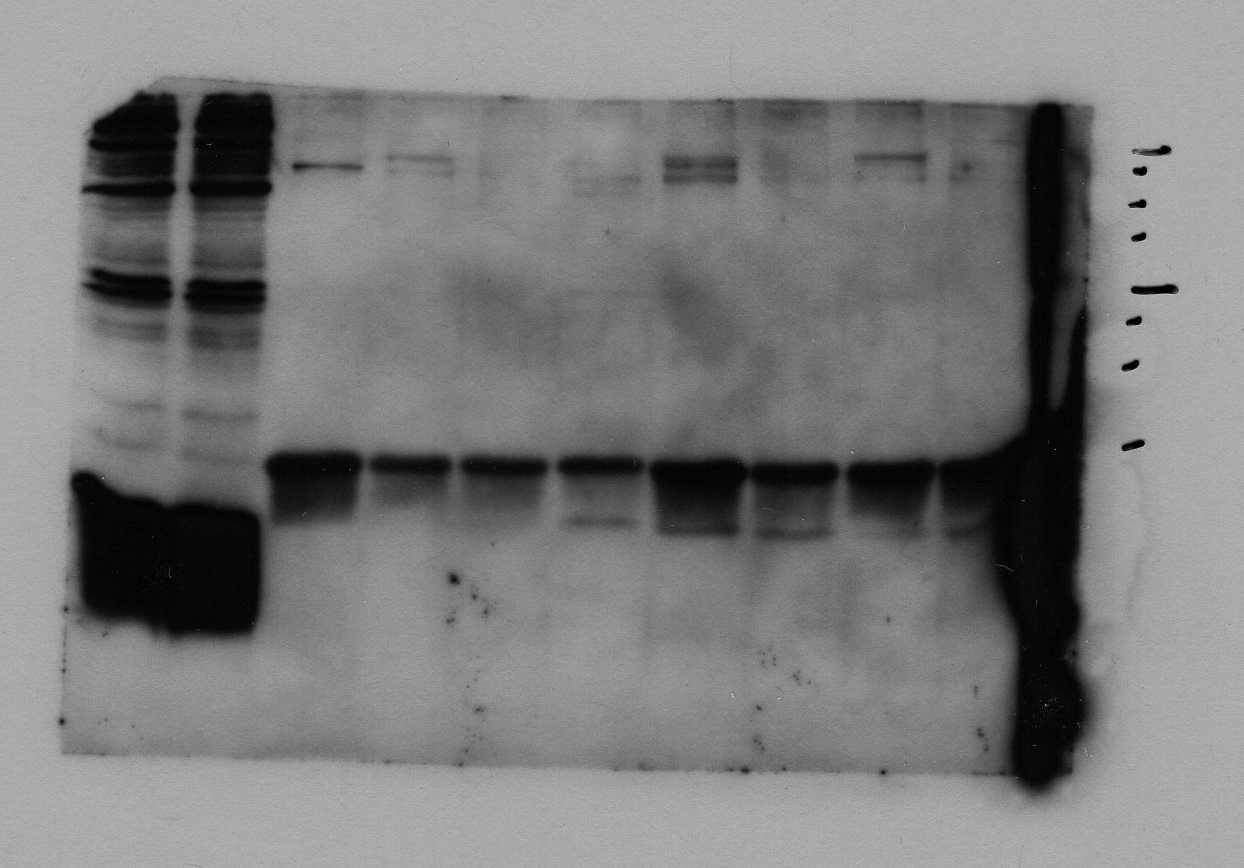

Supplement: Figure 1—source data 3. [file elife-102680-fig1-data3.zip › Figure 1 - Source data 3/Figure 1F_ Source data 3/Pull-down-BM_ATP5i_validation_1020.tif]

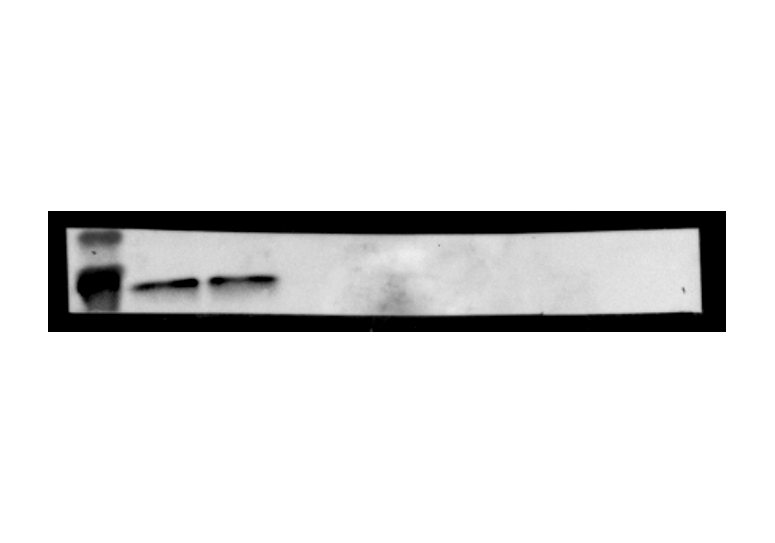

Supplement: Figure 2—source data 2. [file elife-102680-fig2-data2.zip › Figure 2 - Source data 2/Figure 2A_ Source data 2/ATP5I.tif]

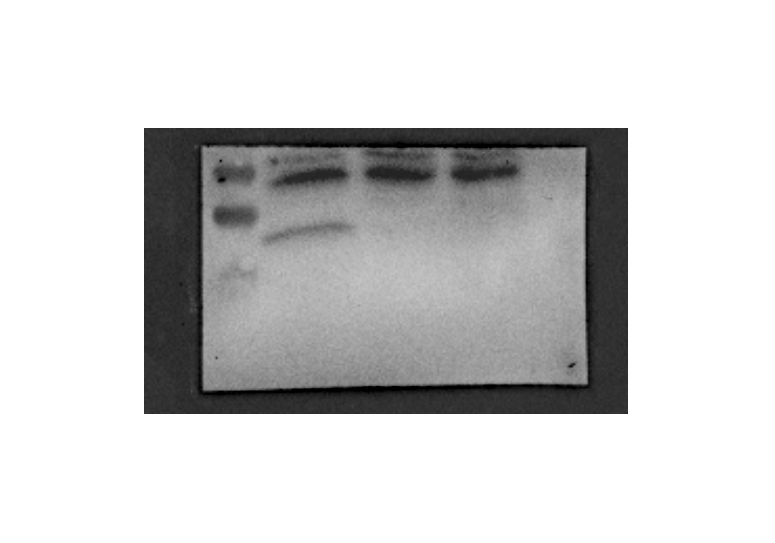

Supplement: Figure 2—source data 2. [file elife-102680-fig2-data2.zip › Figure 2 - Source data 2/Figure 2A_ Source data 2/ATP5L.tif]

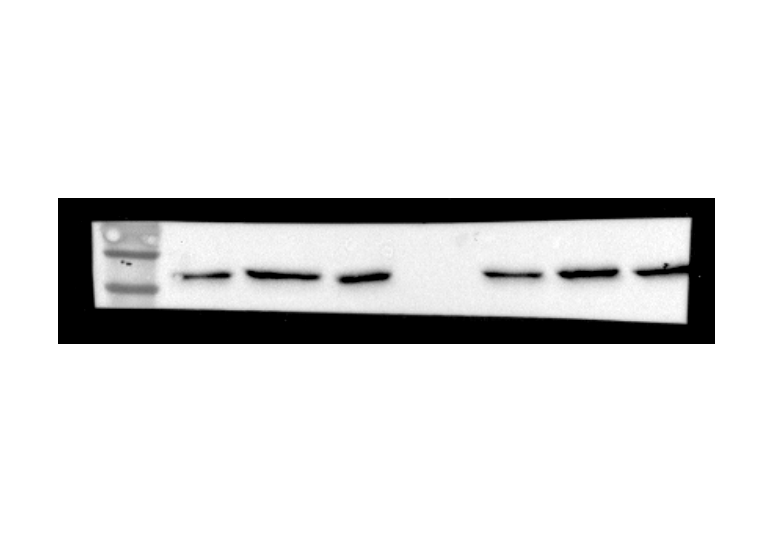

Supplement: Figure 2—source data 2. [file elife-102680-fig2-data2.zip › Figure 2 - Source data 2/Figure 2A_ Source data 2/b-subunit.tif]

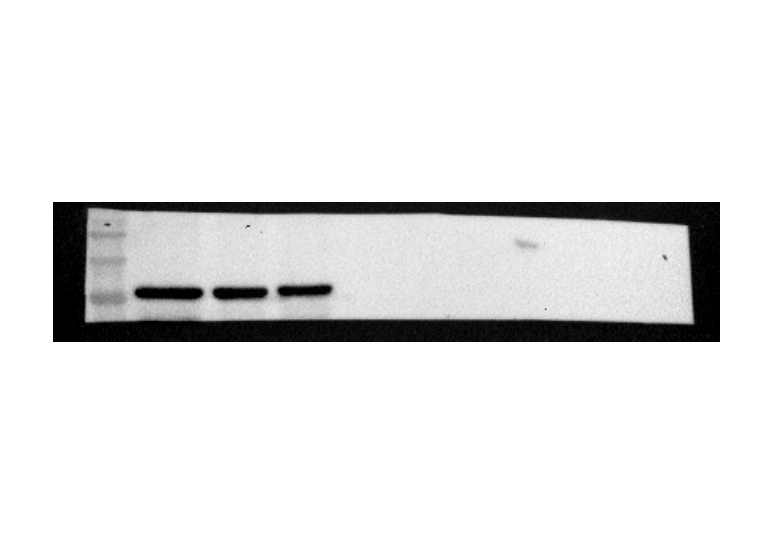

Supplement: Figure 2—source data 2. [file elife-102680-fig2-data2.zip › Figure 2 - Source data 2/Figure 2A_ Source data 2/GAPDH.tif]

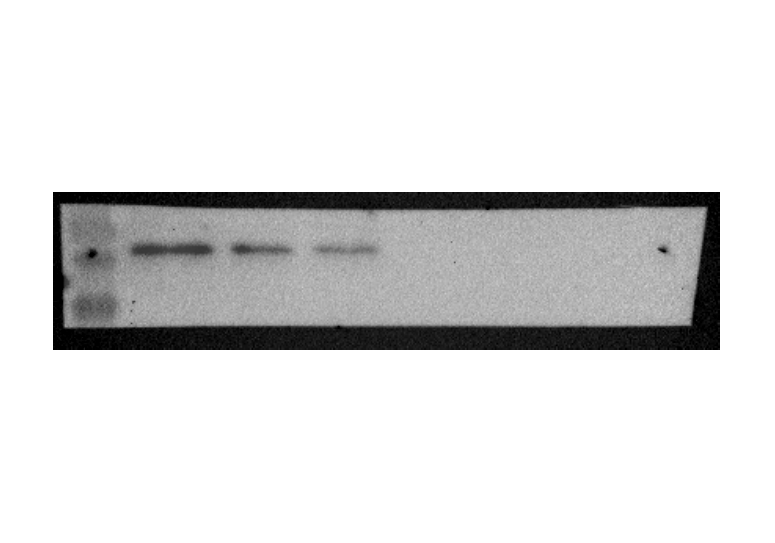

Supplement: Figure 2—source data 2. [file elife-102680-fig2-data2.zip › Figure 2 - Source data 2/Figure 2A_ Source data 2/OSCP.tif]

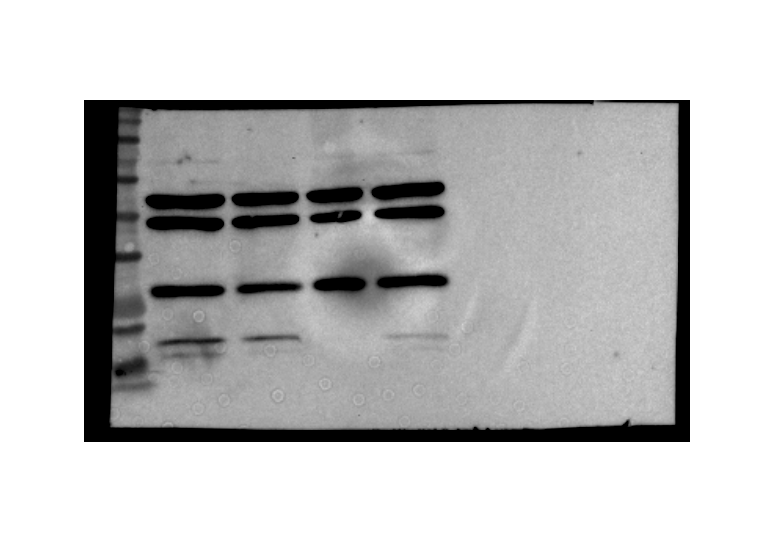

Supplement: Figure 2—source data 2. [file elife-102680-fig2-data2.zip › Figure 2 - Source data 2/Figure 2A_ Source data 2/OXPHOS.tif]

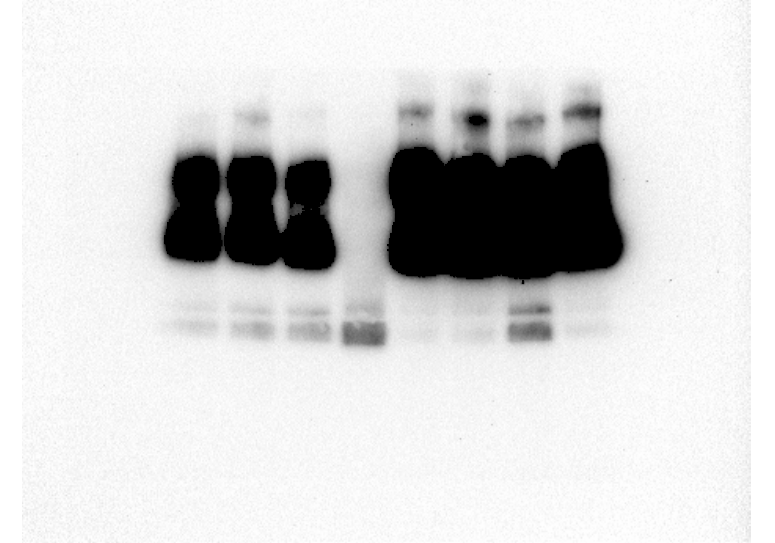

Supplement: Figure 2—source data 2. [file elife-102680-fig2-data2.zip › Figure 2 - Source data 2/Figure 2G_ Source data 2/BN_PAGE_300s.tif]

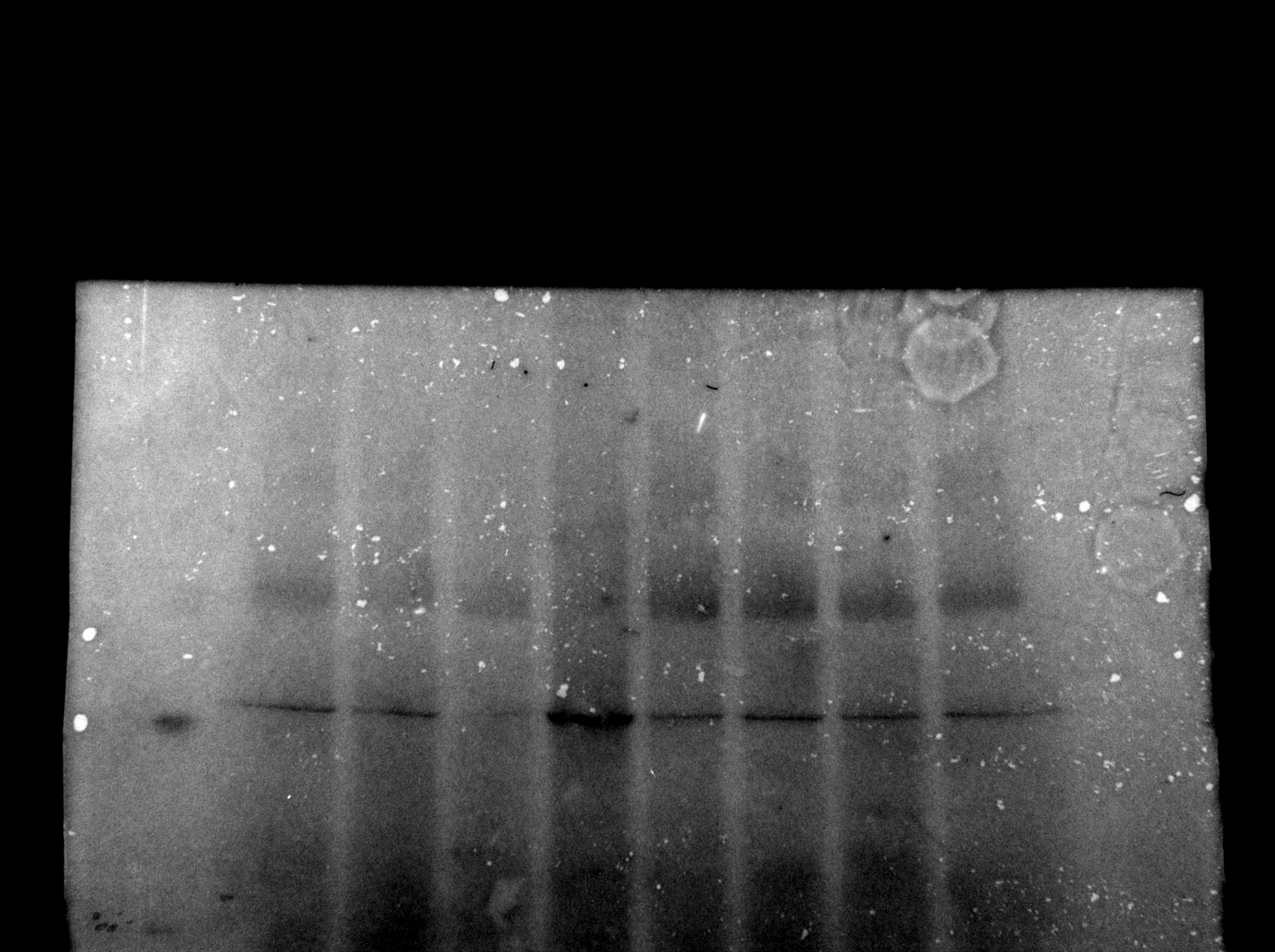

Supplement: Figure 2—source data 2. [file elife-102680-fig2-data2.zip › Figure 2 - Source data 2/Figure 2G_ Source data 2/Ponceau_BN_PAGE.tif]

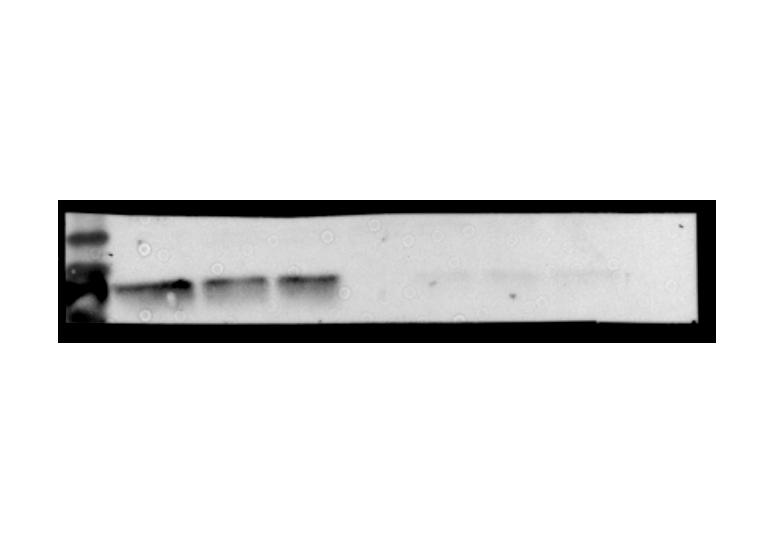

Supplement: Figure 2—figure supplement 1—source data 2. [file elife-102680-fig2-figsupp1-data2.zip › Figure 2 - Figure supplement 1 - Source data 2/Figure 2_ Figure supplement 1_ Source data 2/ATP5I.tif]

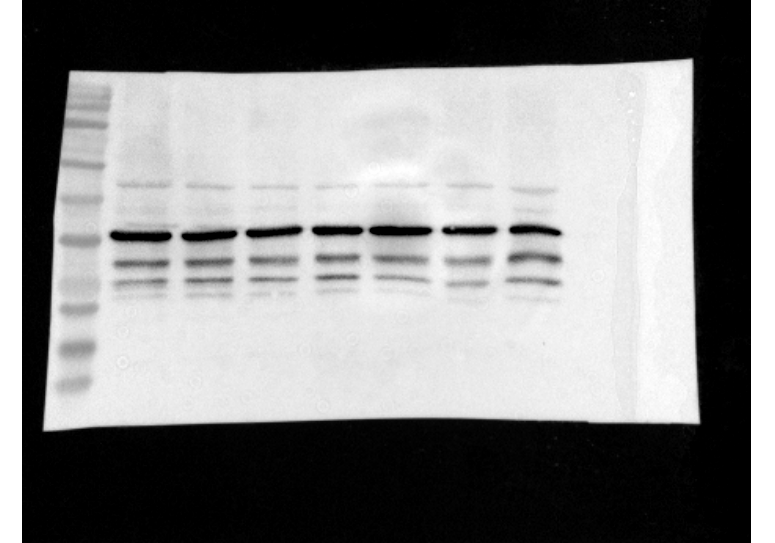

Supplement: Figure 2—figure supplement 1—source data 2. [file elife-102680-fig2-figsupp1-data2.zip › Figure 2 - Figure supplement 1 - Source data 2/Figure 2_ Figure supplement 1_ Source data 2/GAPDH.png]

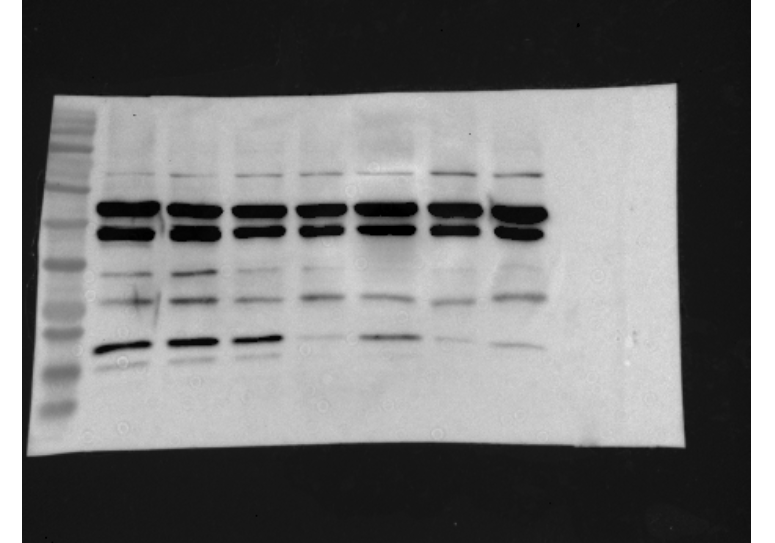

Supplement: Figure 2—figure supplement 1—source data 2. [file elife-102680-fig2-figsupp1-data2.zip › Figure 2 - Figure supplement 1 - Source data 2/Figure 2_ Figure supplement 1_ Source data 2/OXPHOS.png]

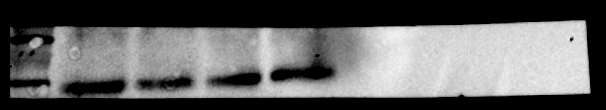

Supplement: Figure 3—source data 2. [file elife-102680-fig3-data2.zip › Figure 3 - Source data 2/Figure 3_ Source data 2/AMPK.tif]

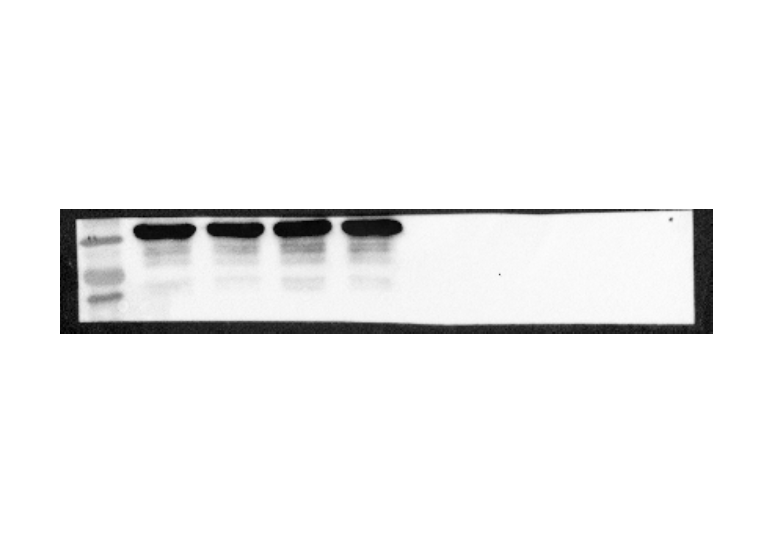

Supplement: Figure 3—source data 2. [file elife-102680-fig3-data2.zip › Figure 3 - Source data 2/Figure 3_ Source data 2/GAPDH.tif]

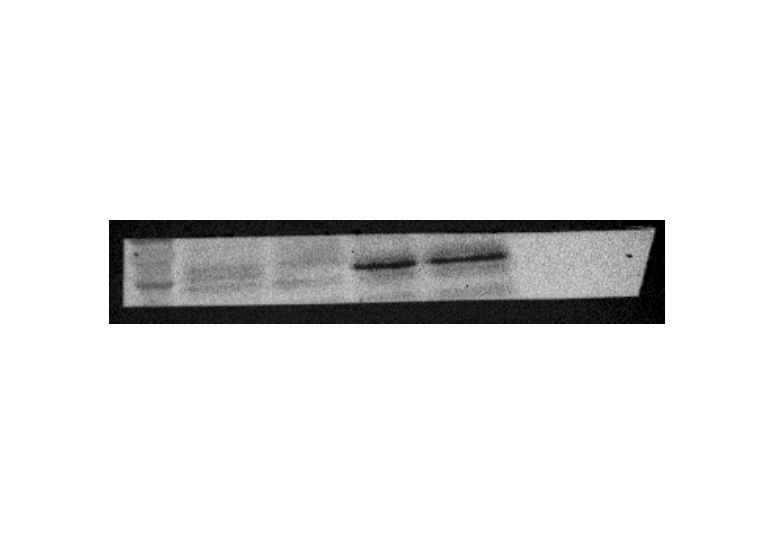

Supplement: Figure 3—source data 2. [file elife-102680-fig3-data2.zip › Figure 3 - Source data 2/Figure 3_ Source data 2/P-AMPK.tif]

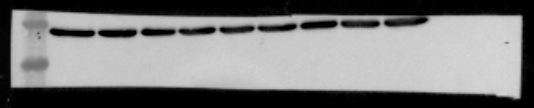

Supplement: Figure 3—figure supplement 1—source data 2. [file elife-102680-fig3-figsupp1-data2.zip › Figure 3 - Figure supplement 1 - Source data 2/Figure 3_ Figure supplement 1_ Source data 2/ACTIN.tif]

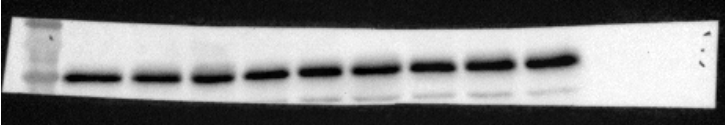

Supplement: Figure 3—figure supplement 1—source data 2. [file elife-102680-fig3-figsupp1-data2.zip › Figure 3 - Figure supplement 1 - Source data 2/Figure 3_ Figure supplement 1_ Source data 2/AMPK.tif]

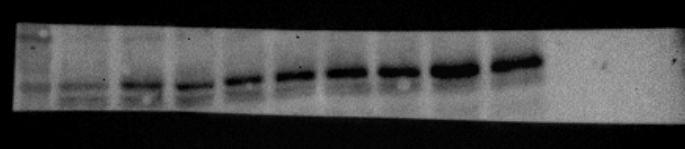

Supplement: Figure 3—figure supplement 1—source data 2. [file elife-102680-fig3-figsupp1-data2.zip › Figure 3 - Figure supplement 1 - Source data 2/Figure 3_ Figure supplement 1_ Source data 2/P-AMPK.tif]

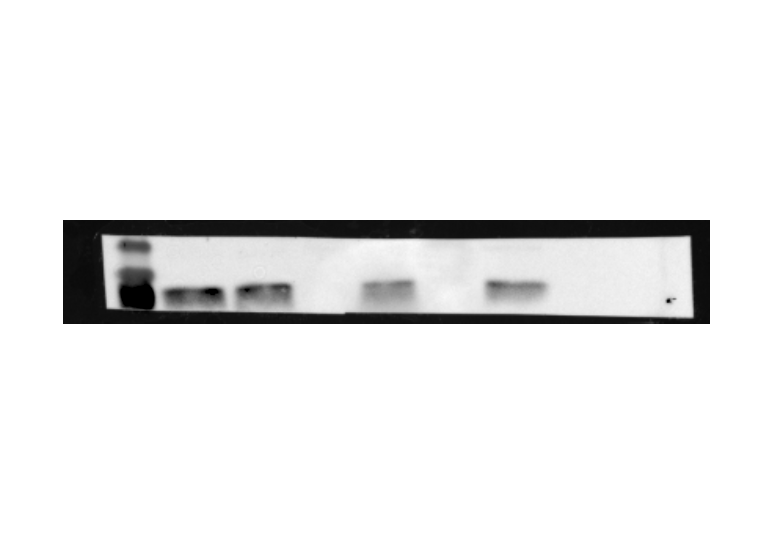

Supplement: Figure 4—source data 2. [file elife-102680-fig4-data2.zip › Figure 4 - Source data 2/Figure 4_ Source data 2/ATP5I.tif]

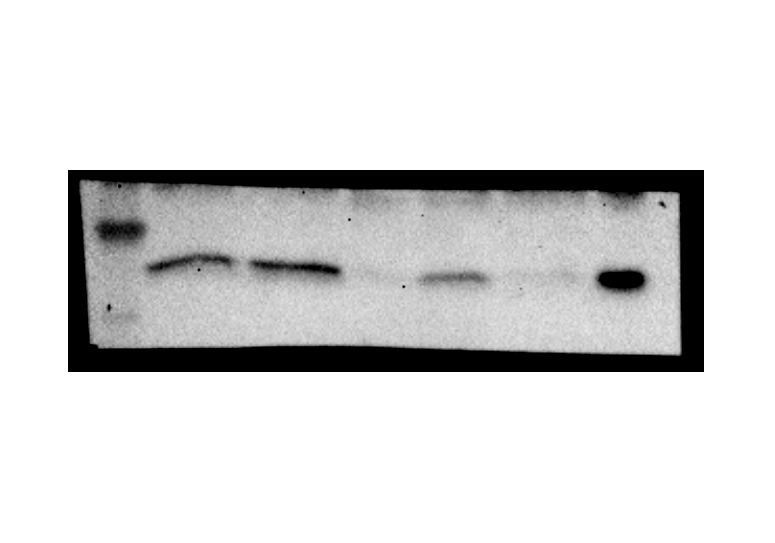

Supplement: Figure 4—source data 2. [file elife-102680-fig4-data2.zip › Figure 4 - Source data 2/Figure 4_ Source data 2/ATP5L.tif]

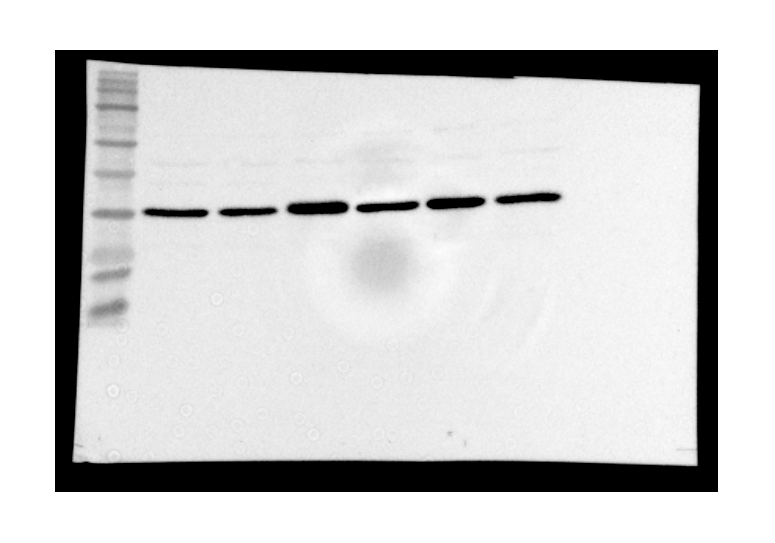

Supplement: Figure 4—source data 2. [file elife-102680-fig4-data2.zip › Figure 4 - Source data 2/Figure 4_ Source data 2/GAPDH.tif]

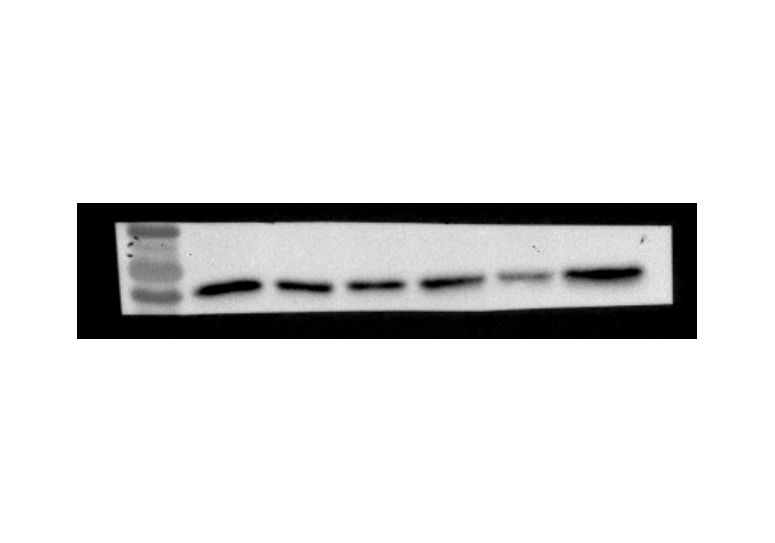

Supplement: Figure 4—source data 2. [file elife-102680-fig4-data2.zip › Figure 4 - Source data 2/Figure 4_ Source data 2/OSCP.tif]

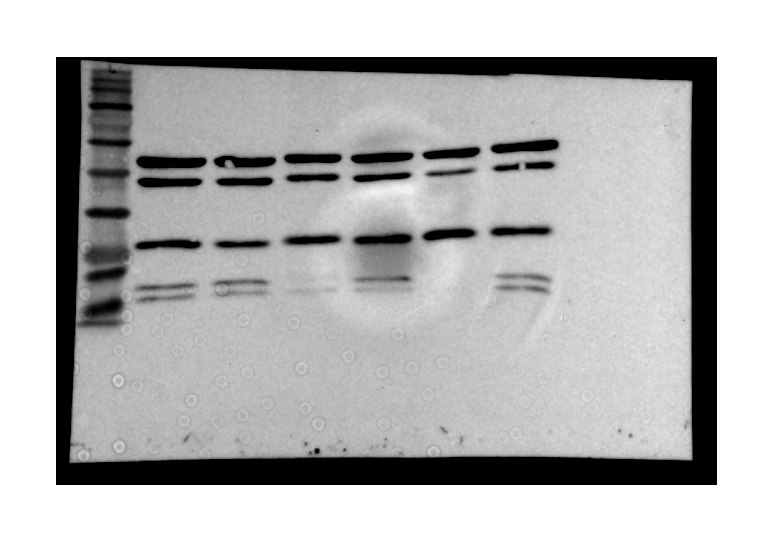

Supplement: Figure 4—source data 2. [file elife-102680-fig4-data2.zip › Figure 4 - Source data 2/Figure 4_ Source data 2/OXPHOS.tif]

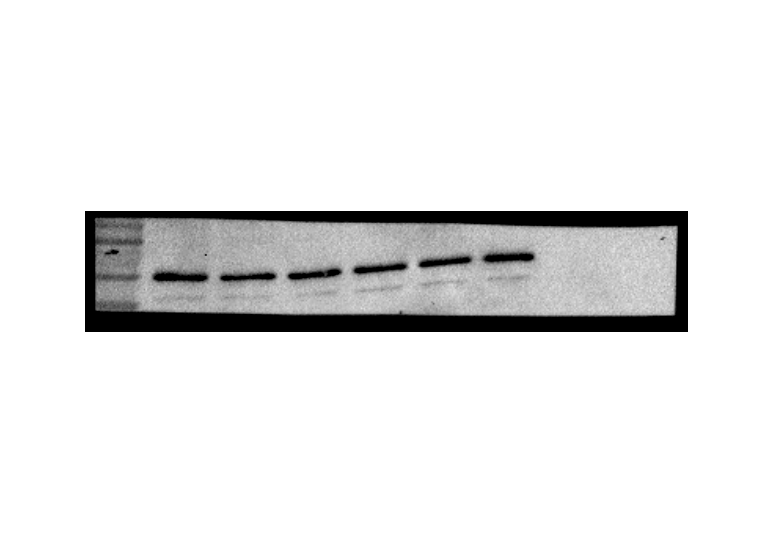

Supplement: Figure 5—source data 2. [file elife-102680-fig5-data2.zip › Figure 5 - Source data 2/Figure 5_ Source data 2/AMPK.tif]

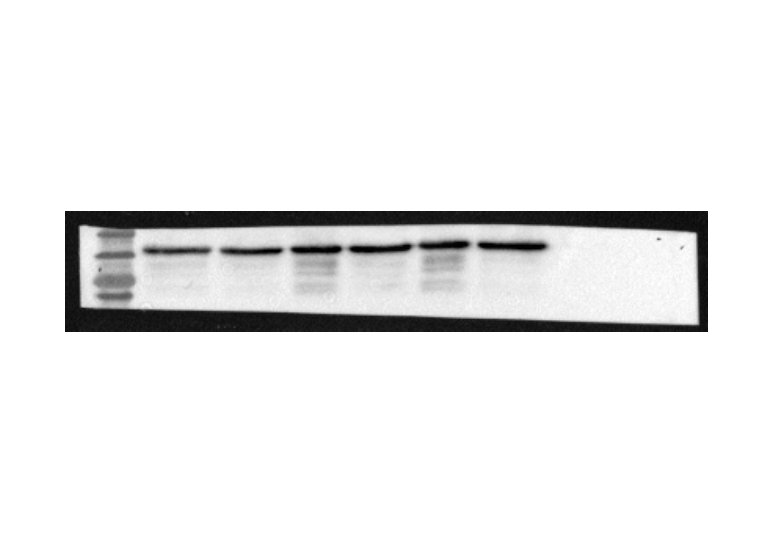

Supplement: Figure 5—source data 2. [file elife-102680-fig5-data2.zip › Figure 5 - Source data 2/Figure 5_ Source data 2/GAPDH.tif]

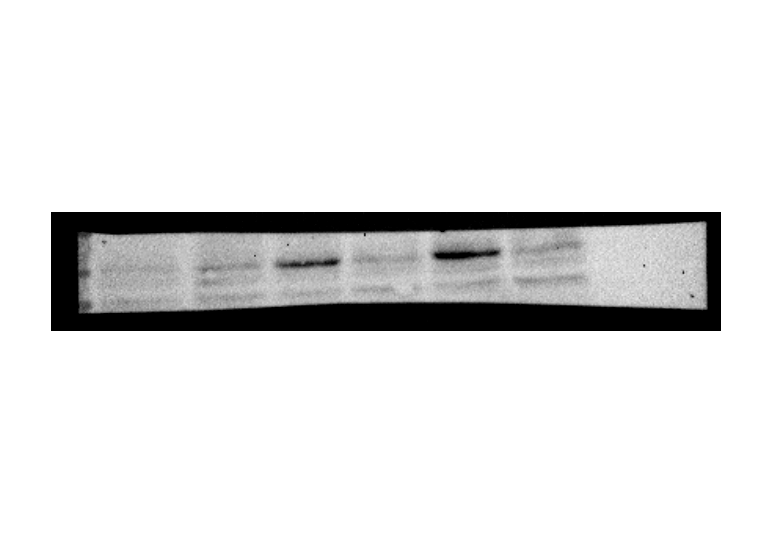

Supplement: Figure 5—source data 2. [file elife-102680-fig5-data2.zip › Figure 5 - Source data 2/Figure 5_ Source data 2/P-AMPK.tif]
