# Supplementary material for: The role of ATP synthase subunit e (ATP5I) in mediating the metabolic and antiproliferative effects of metformin in cancer cells
Source: eLife. 2026 May 15;13:RP102680. doi: 10.7554/eLife.102680 (PMC13179060; doi:10.7554/eLife.102680)
Supplement: Figure 2—source data 1. [file elife-102680-fig2-data1.zip › Figure 2 - Source data 1/Figure 2A_Source data 1.pdf]

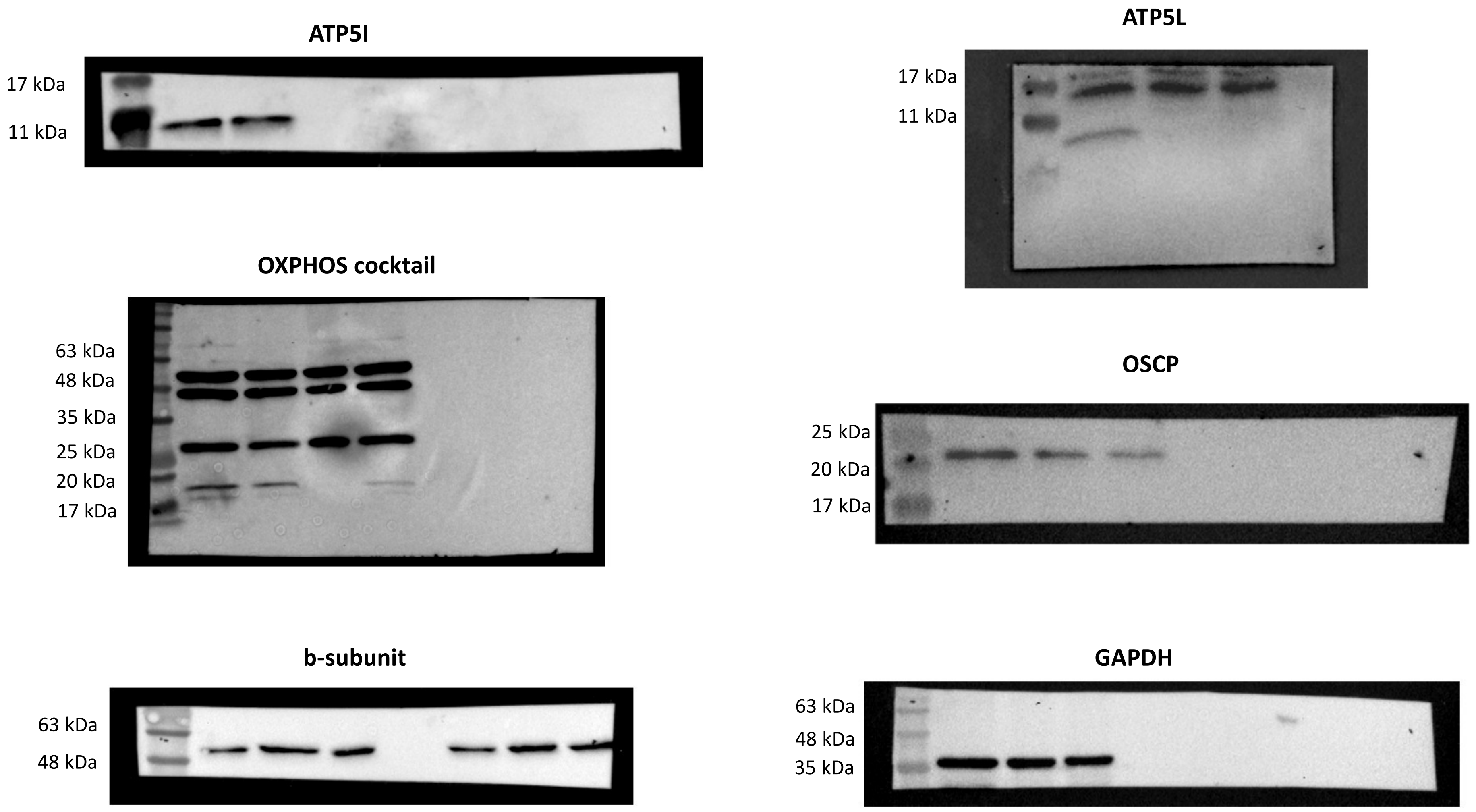

Figure 2A, Source Data 1. Original membranes corresponding to Figure 2A. For ATP5I and OXPHOS cocktail (originally from the preprint), lane 1 corresponds to non-infected cells, lane 2 to GFP control cells, lane 3 to ATP5I guide #1, and lane 4 to ATP5I guide #2. For ATP5L, OSCP, b subunit, and GAPDH, lane 1 corresponds to GFP control, lane 2 to ATP5I guide #1, and lane 3 to ATP5I guide #2. For the b subunit blot, lanes 4–6 correspond to the same conditions repeated as biological replicate N = 2. Apparent molecular weight positions are indicated using the annotated blue prestained protein marker.
