# Supplementary material for: The role of ATP synthase subunit e (ATP5I) in mediating the metabolic and antiproliferative effects of metformin in cancer cells
Source: eLife. 2026 May 15;13:RP102680. doi: 10.7554/eLife.102680 (PMC13179060; doi:10.7554/eLife.102680)
Supplement: Figure 2—source data 1. [file elife-102680-fig2-data1.zip › Figure 2 - Source data 1/Figure 2G_Source data 1.pdf]

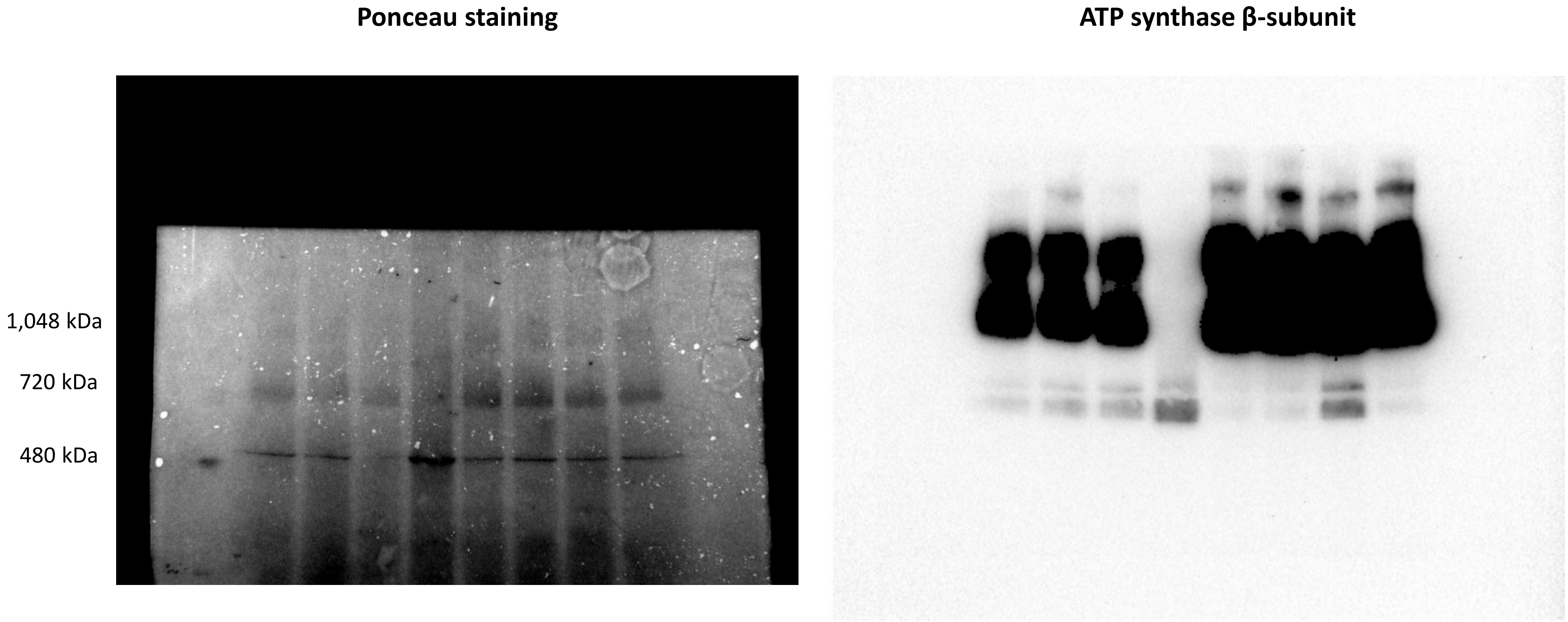

Figure 2G, Source Data 1. Original membrane corresponding to Figure 2G. Lanes 1–3 correspond to U2OS cells untreated, treated with metformin (16 h, 10 mM), and treated with metformin (72 h, 10 mM), respectively. Lane 4 corresponds to ATP5I knockout KP-4 cells, and lanes 5–8 correspond to KP-4 cells under the following conditions: untreated, metformin (16 h, 10 mM), metformin (72 h, 10 mM), and rotenone (16 h, 50 nM), respectively. An annotated Ponceau staining image (Figure 2, figure supplement 2) is provided to indicate the relevant labeled bands. Molecular weight marker positions are annotated manually to indicate the apparent size of the detected bands.
