# Supplementary material for: The role of ATP synthase subunit e (ATP5I) in mediating the metabolic and antiproliferative effects of metformin in cancer cells
Source: eLife. 2026 May 15;13:RP102680. doi: 10.7554/eLife.102680 (PMC13179060; doi:10.7554/eLife.102680)
Supplement: Figure 2—figure supplement 1—source data 1. [file elife-102680-fig2-figsupp1-data1.zip › Figure 2 - Figure supplement 1 - Source data 1/Figure 2_Figure supplement 1_Source data 1.pdf]

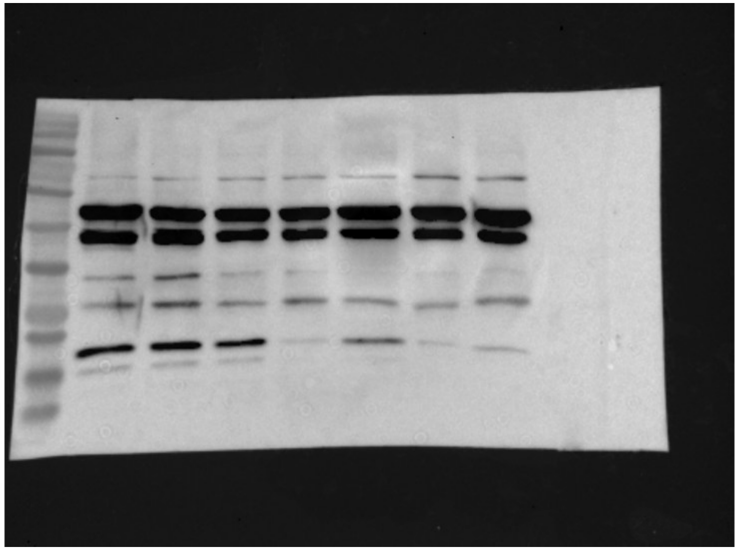


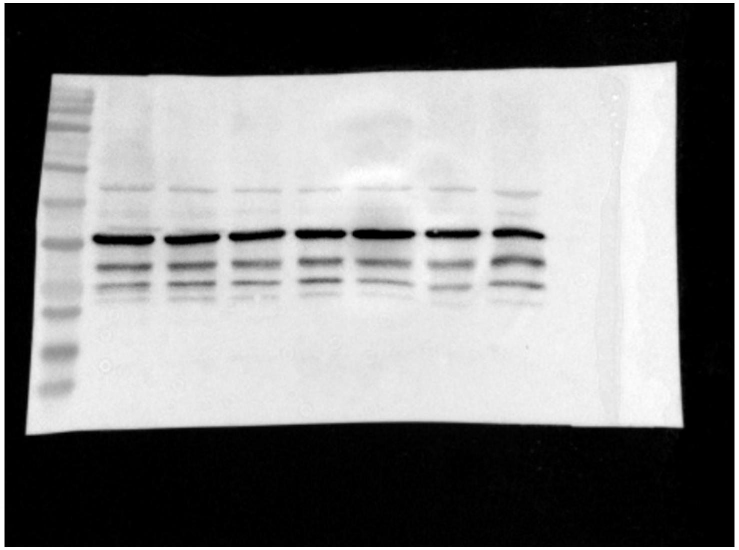


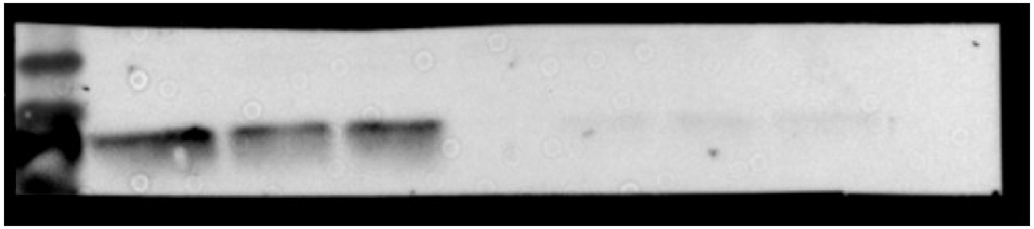


**Figure 2, Figure supplement 1, Source Data 1.** Original membranes corresponding to Figure 2, Figure supplement 1. Lane 1 corresponds to non-infected cells. Lanes 2–3 correspond to two clones of GFP control cells. Lanes 4–5 correspond to two clones of ATP5I guide #1 cells, and lanes 6–7 correspond to two clones of ATP5I guide #2 cells. Apparent molecular weight positions are indicated using the annotated blue prestained protein marker.
