# Supplementary material for: The role of ATP synthase subunit e (ATP5I) in mediating the metabolic and antiproliferative effects of metformin in cancer cells
Source: eLife. 2026 May 15;13:RP102680. doi: 10.7554/eLife.102680 (PMC13179060; doi:10.7554/eLife.102680)
Supplement: Figure 2—figure supplement 2—source data 1. [file elife-102680-fig2-figsupp2-data1.zip › Figure 2 - Figure supplement 2 - Source data 1/Figure 2_Figure supplement 2_Source data 1.pdf]

Ponceau staining

1,048 kDa

720 kDa

480 kDa

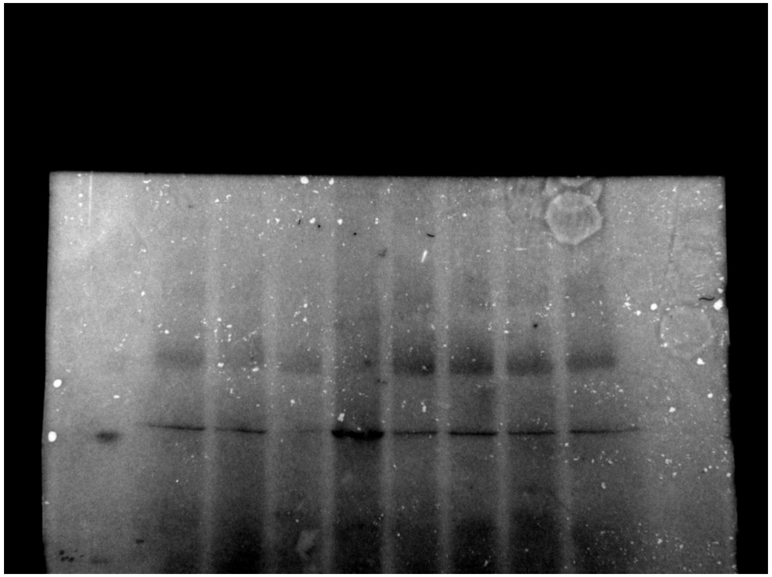

Figure 2, Figure supplement 2, Source Data 1. Original Ponceau-stained membrane corresponding to Figure 2G. Lanes 1–3 correspond to U2OS cells untreated, treated with metformin (16 h, 10 mM), and treated with metformin (72 h, 10 mM), respectively. Lane 4 corresponds to ATP5I knockout KP-4 cells, and lanes 5–8 correspond to KP-4 cells under the following conditions: untreated, metformin (16 h, 10 mM), metformin (72 h, 10 mM), and rotenone (16 h, 50 nM), respectively. Molecular weight marker positions are annotated manually to indicate the apparent size of the detected bands.
