# Supplementary material for: The role of ATP synthase subunit e (ATP5I) in mediating the metabolic and antiproliferative effects of metformin in cancer cells
Source: eLife. 2026 May 15;13:RP102680. doi: 10.7554/eLife.102680 (PMC13179060; doi:10.7554/eLife.102680)
Supplement: Figure 3—source data 1. [file elife-102680-fig3-data1.zip › Figure 3 - Source data 1/Figure 3_Source data 1.pdf]

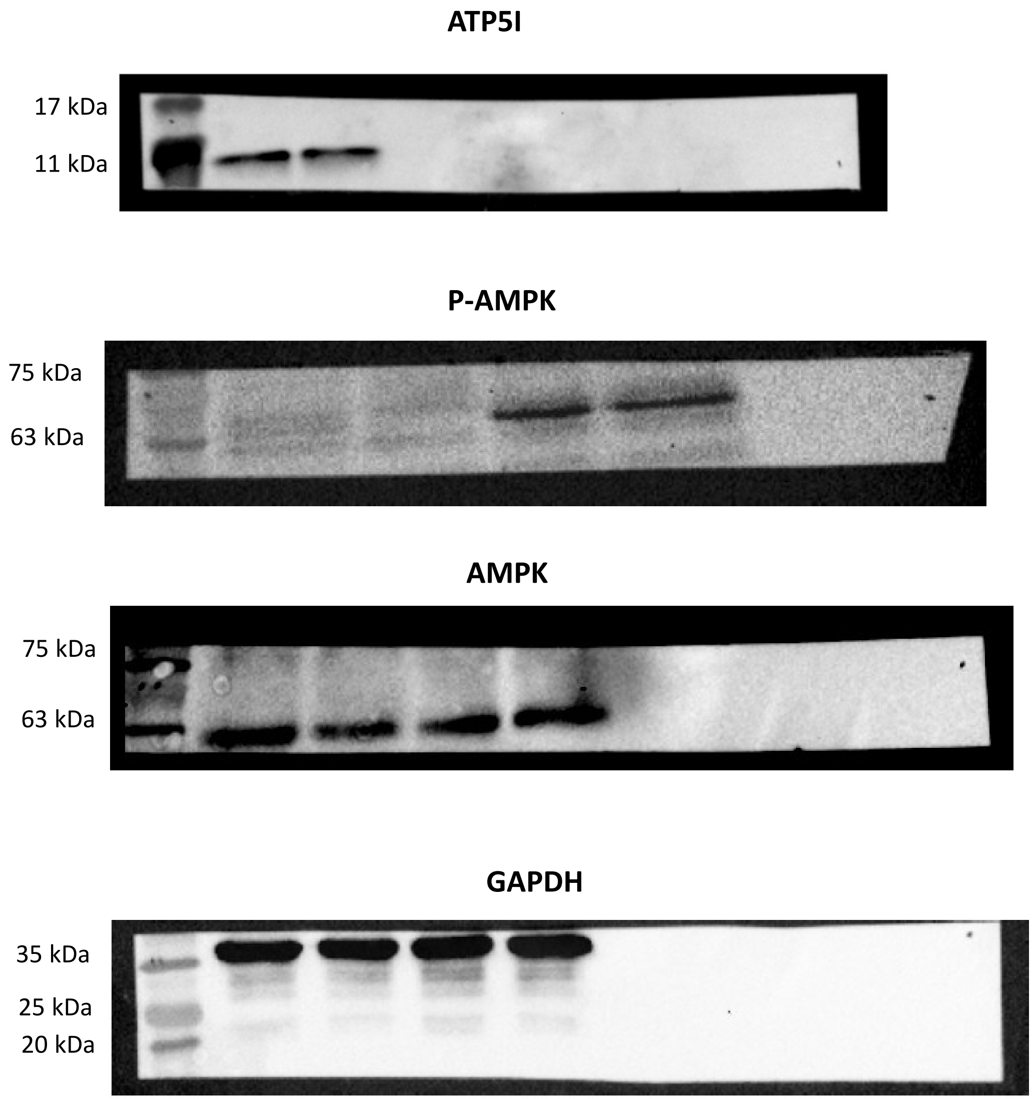

Figure 3, Source Data 1. Original membranes corresponding to Figure 3. Lane 1 corresponds to non-infected cells, lane 2 to GFP control cells, lane 3 to ATP5I guide #1, and lane 4 to ATP5I guide #2 respectively. Apparent molecular weight positions are indicated using the annotated blue prestained protein marker.
