# Supplementary material for: The role of ATP synthase subunit e (ATP5I) in mediating the metabolic and antiproliferative effects of metformin in cancer cells
Source: eLife. 2026 May 15;13:RP102680. doi: 10.7554/eLife.102680 (PMC13179060; doi:10.7554/eLife.102680)
Supplement: Figure 3—figure supplement 1—source data 1. [file elife-102680-fig3-figsupp1-data1.zip › Figure 3 - Figure supplement 1 - Source data 1/Figure 3_Figure supplement 1_Source data 1.pdf]

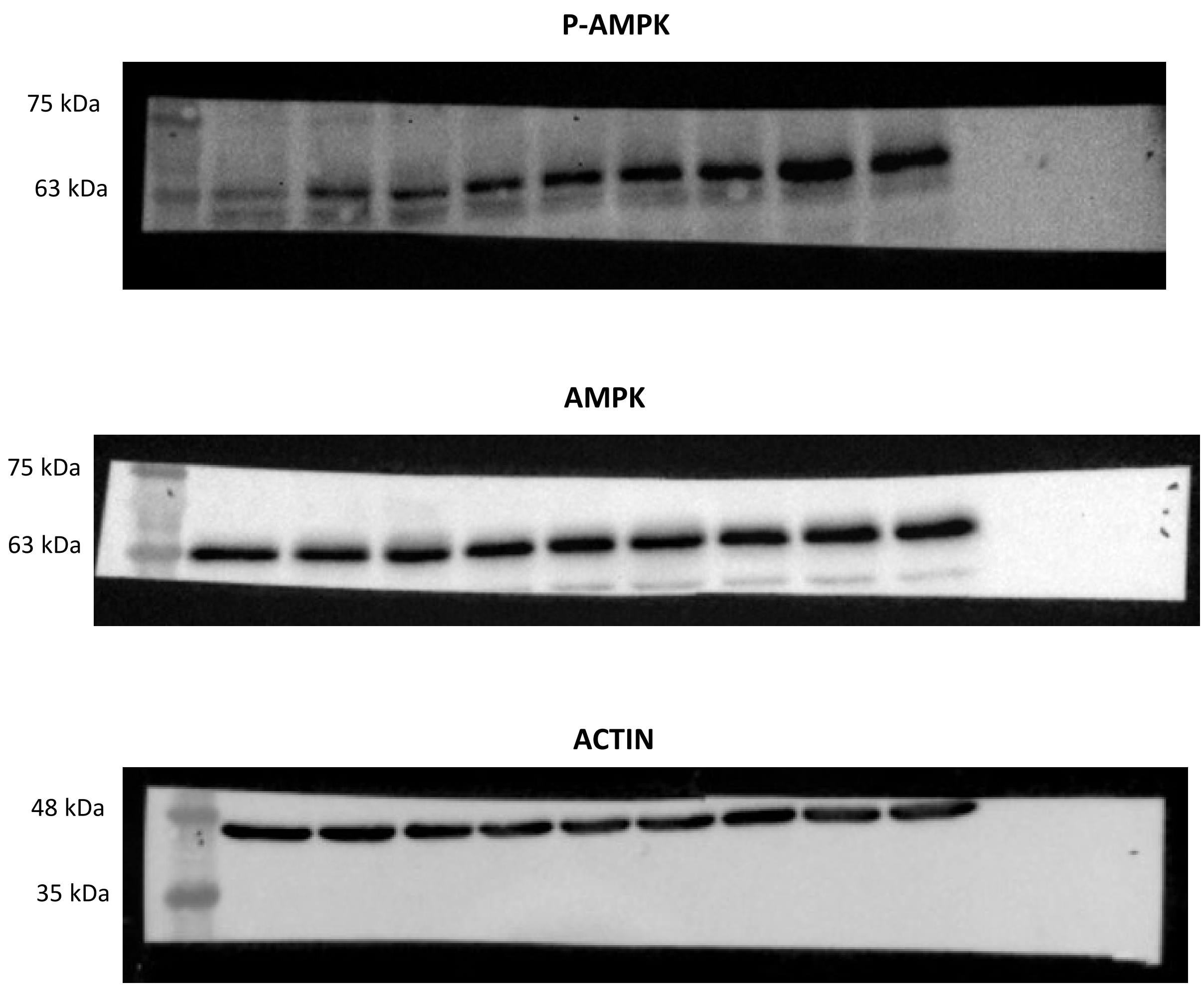

Figure 3, Figure Supplement 1, Source Data 1. Original membranes corresponding to Figure 3, Figure Supplement 1. Lanes 1–3 correspond to GFP control cells untreated, treated with 2.5 mM metformin, and treated with 5 mM metformin, respectively. Lanes 4–6 correspond to the same conditions for ATP5I guide #1 cells. Lanes 7–9 correspond to the same conditions for ATP5I guide #2 cells. Apparent molecular weight positions are indicated using the annotated blue prestained protein marker.
