# Supplementary material for: The role of ATP synthase subunit e (ATP5I) in mediating the metabolic and antiproliferative effects of metformin in cancer cells
Source: eLife. 2026 May 15;13:RP102680. doi: 10.7554/eLife.102680 (PMC13179060; doi:10.7554/eLife.102680)
Supplement: Figure 4—source data 1. [file elife-102680-fig4-data1.zip › Figure 4 - Source data 1/Figure 4_Source data 1.pdf]

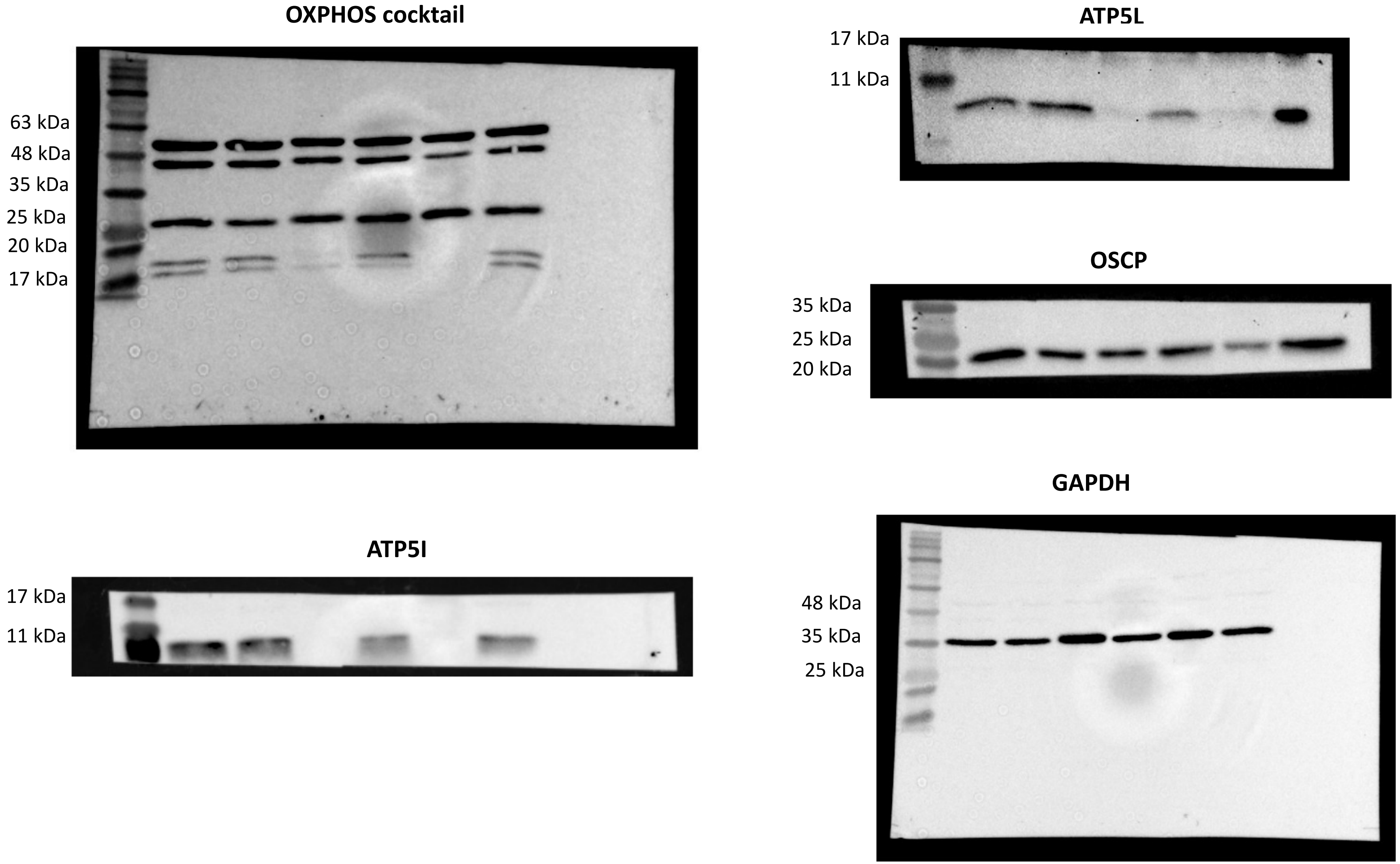

Figure 4, Source Data 1. Original membranes corresponding to Figure 4. Lanes 1–2 correspond to GFP control cells without or with exogenous ATP5I expression, respectively. Lanes 3–4 correspond to ATP5I guide #1 cells without or with exogenous ATP5I expression, respectively. Lanes 5–6 correspond to ATP5I guide #2 cells without or with exogenous ATP5I expression, respectively. Apparent molecular weight positions are indicated using the annotated blue prestained protein marker.
